# Influence of C3′- and C4′-substitutions on fluorescence, crystal packing, and physicochemical properties of flavonol

**DOI:** 10.1039/d5ra05790f

**Published:** 2025-09-30

**Authors:** Oleksii O. Demidov, Ludmila V. Chepeleva, Svitlana V. Shishkina, Eugene S. Gladkov, Alexander Kyrychenko, Rostyslav P. Linnik, Alexander D. Roshal

**Affiliations:** a Institute of Chemistry, V.N. Karazin Kharkiv National University 4 Svobody Sq. Kharkiv 61022 Ukraine a.v.kyrychenko@karazin.ua; b Institute of Functional Materials Chemistry, State Scientific Institution “Institute for Single Crystals” of National Academy of Sciences of Ukraine 60 Nauky Ave 61072 Kharkiv Ukraine; c Institute of Organic Chemistry, NAS of Ukraine 5 Akademik Kukhar Str. Kyiv 02066 Ukraine; d - Taras Shevchenko National University of Kyiv, Analytical Chemistry Department 64/13 Volodymyrska Str. Kyiv 01601 Ukraine

## Abstract

Natural flavonols exhibit a wide range of pharmacological activities and possess unique dual ESIPT (Excited-State Intramolecular Proton Transfer) fluorescence, making them sensitive to microenvironments. This sensitivity allows for the detection of metal ions, anions, small ligands, and biomacromolecules. However, the diversity in their structure, including the number and position of hydroxyl groups and potential chemical modifications, complicates the relationship between structure and fluorescence, posing challenges for their practical use as fluorescent probes. In this study, we focus on fine-tuning the ESIPT fluorescence, crystal packing, physicochemical properties, and ADMET (Absorption, Distribution, Metabolism, Excretion, and Toxicity) characteristics of a series of flavonols. We achieve this by introducing hydroxy, methoxy and benzyl groups at the C3′ and C4′ positions of the 2-phenyl side ring. The photophysical properties of the synthesized flavonols were systematically examined by UV-vis and fluorescence measurements in terms of their structure–property relationship. Our findings indicate that the nature and position of the substituent groups in flavonols can significantly influence their crystal packing in the solid state, tuning contributions of intra- and intermolecular hydrogen bonding and the ESIPT behavior. Lastly, through fluorescence titration and molecular docking calculations, we explored how the introduction of a bulky benzyl moiety and its alteration between C3′ and C4′ positions can influence the binding interactions of flavonols with β-glucosidases. We believe our findings shed light on the structure–fluorescence relationship in flavonols and open up new opportunities for the design of innovative flavonol-based probes.

## Abstract 2

Fine-tuning the ESIPT fluorescence, crystal packing, and physicochemical properties of a series of flavonols was achieved by introducing hydroxy, methoxy and benzyloxy groups at the C3′ and C4′ positions of the 2-phenyl side ring.

## Graphical abstract

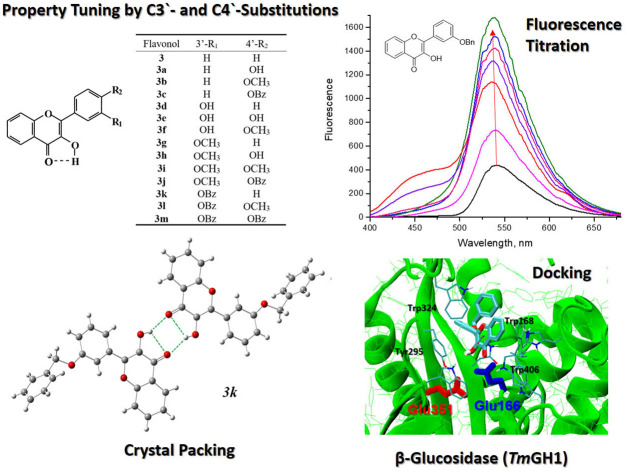

## Introduction

Flavonols are widely distributed compounds found in nature, primarily as secondary metabolites of plants and fungi.^1^ These substances possess a variety of biological effects, making them of great interest to pharmaceutical researchers.^2^ They are known for their strong antioxidant and cancerostatic properties, and their P-vitamin activity.^1,2^ Additionally, flavonols serve as effective models for studying intramolecular excited-state proton transfer (ESIPT) processes.^3–8^ The phototautomerization of flavonols leads to the formation of two distinct emission bands in fluorescence spectra. The intensity ratio of these bands—corresponding to the initial excited form and the phototautomer — depends not only on the structure of the flavonol compounds but also on external factors like the polarity of the surrounding environment and specific interactions with solvent molecules.^3,9–14^ Due to their properties, flavonols can serve as sensors in biochemical and medico-biological studies.^9,15,16^ The 7-hydroxy group in flavones can undergo photodissociation, resulting in fluorescent anions when the corresponding flavones are in an excited state.^17,18^ In the case of 7-hydroxyflavonols, photodissociation occurs alongside excited-state intramolecular proton transfer (ESIPT), leading to the formation of more complex protolytic forms known as anion-tautomers.^19^ The fluorescence of mentioned flavonol molecules is utilized to study micelles and cell membranes.^20–22^ Additionally, flavonols possess one to four complexation centers, enabling them to function as fluorescent indicators for detecting metal ions.^21,23,24^ They also may act as ligands that enhance the efficient extraction of metal ions into the organic phase.^25^

All the manifestations of flavonols' reactivity share common characteristics in terms of their spectral and pharmacological effects. These changes in flavonol structures, such as photodissociation, photoisomerization, complex formation, and interactions with surrounding molecules, typically involve the chromone fragment of the molecule.^24^ The effects of the side phenyl ring and its substituents on the reactivity, spectral, and pharmacological properties have not been thoroughly studied.

The introduction of strong electron-donating and electron-accepting groups, such as alkylamino substituents or nitro groups, into the side ring can result in significant interfragment charge transfer (ICT) between the phenyl and chromone fragments. This transfer leads to the appearance of new long-wavelength absorption bands in the spectra, attributed to the charge–transfer transition, along with changes in the fluorescence characteristics. Natural and many synthetic flavonols typically contain less effective electron-donating substituents, such as hydroxy or various alkoxy groups.^8^ Their impact on the spectral properties of flavonols, as previously noted, has not been thoroughly studied. Additionally, our recent studies indicate that flavonols with lipophilic alkoxy substituents on the side phenyl ring can effectively bind to hydrolase enzymes, such as glucosidases, and inhibit their catalytic activity.^21,26^

Physicochemical and pharmacological properties of flavonols have complex interplay on the position and number of hydroxyl groups, as well as their chemical modifications.^2^ Therefore, in this study, we focus on some flavonols with various combinations of substituents in the C3′ and C4′ positions of the side benzene ring. The introduction of methoxy and benzyl groups and altering their positions aimed to establish the relationship between the structure and fluorescent properties of flavonols. Our study demonstrates how the nature and position of substituents in the side ring affect the structure of flavonol molecules in both gas and crystalline phases. It also discusses how these factors influence the position of bands in absorption spectra, as well as the position and intensity ratio of emission bands for both the original and phototautomeric forms in fluorescence spectra. Finally, we consider how peripheral phenyl ring substitutions can tune the binding interactions with proteins.

## Experimental section

### Chemistry

**Scheme 1:**
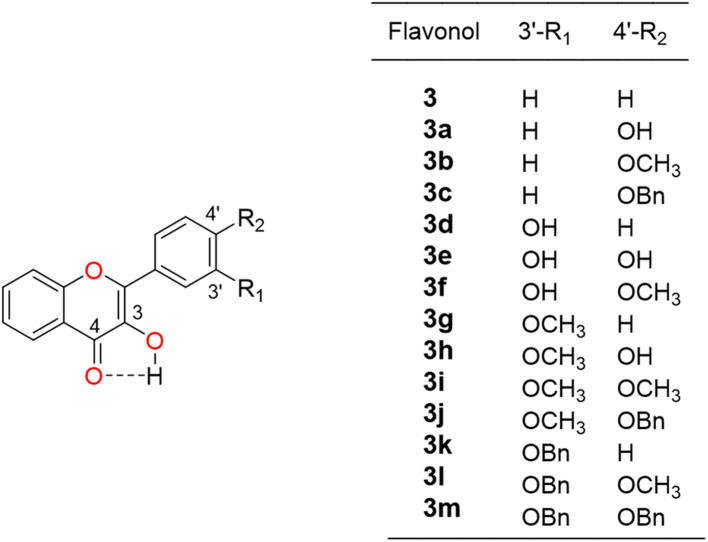
Structure of model flavonol 3 and abbreviations for its derivatives 3a–m.

To study the effect of the C3′ and C4′ substitutions in the flavonol's side benzene ring on fluorescence, crystal packing, physicochemical and ADMET properties the following flavonols were considered (Scheme 1).

**Scheme 2:**
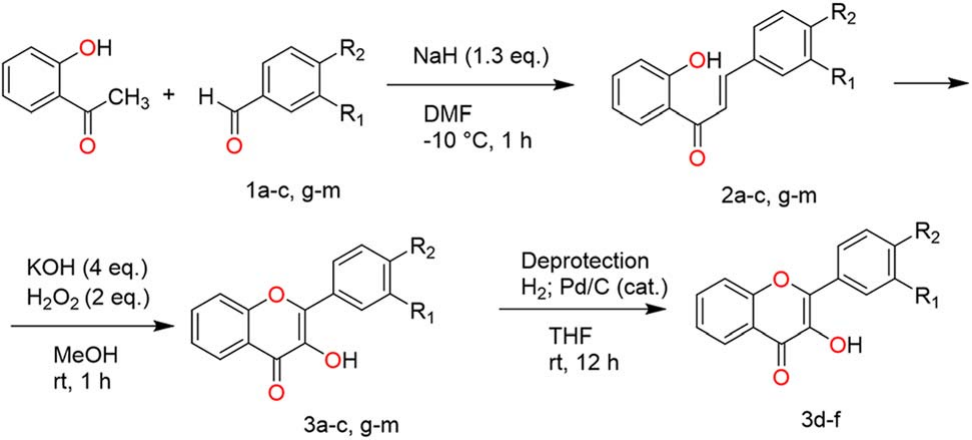
Synthesis of flavonols (3-hydroxy-4H-chromen-4-ones) 3a–m.

Structure of unsubstituted flavonol 3 and synthesized flavonol derivatives 3a–m are summarized in Scheme 1. Unsaturated ketones (2a–c, g–m), used as precursors, were obtained by a modified known methods (Scheme 2).^27,28^ Preliminary spectral analysis was provided by Enamine Ltd (Ukraine). All solvents and reagents were commercial grade and, if required, purified in accordance with the standard procedures.

### General procedure of synthesis of chalcones (2a–c, g–m)

To a suspension of NaH (5.2 mmol, 60% dispersion in mineral oil) in DMF (20 mL), under a nitrogen atmosphere at 0 °C added drop wisely 2′-hydroxyacetophenone (4 mmol). The resulting mixture stirred at 0 °C for 10 min. A solution of corresponding benzaldehyde (4 mmol) in DMF (10 mL) was added drop wisely in the mixture and stirred at room temperature for 2 hours. Therefore, the mixture was acidified drop wisely with glacial acetic acid until pH 4. After filtration the precipitate washed with MeOH (10 mL). The resulting solid was concentrated under vacuum to obtain a crud product with uses in next stage without purification.

### General procedure of synthesis of flavonols (3a–c, g–m)

To a suspension of chalcone 2a–c, g–m (6 mmol) in MeOH (30 mL) was added KOH (15 mmol), the reaction mixture stirred at −15 °C for 10 min. Then 30% H_2_O_2_ (18 mmol) was drop wisely added in the mixture and stirred at rt for 2.5 hours. Therefore, the mixture was acidified drop wisely with glacial acetic acid until pH 2. After filtration the precipitate washed with MeOH (10 mL). The resulting solid was concentrated under vacuum to obtain a pure product. The synthesis scheme is summarized in Scheme 2 and Table 1.

**Table 1:** Synthesized flavonols (3-hydroxy-4H-chromen-4-ones) 3a–m

Flavonol	3′ (*R*_1_)	4′ (*R*_2_)	Starting material	Solvent	Yield, %
3a	H	OH	2a	MeOH	80
3b	H	OCH_3_	2b^28^	MeOH	78
3c	H	OBn	2c	MeOH	73
3d	OH	H	3k	THF	85
3e	OH	OH	3l	THF	76
3f	OH	OCH_3_	3m	THF	90
3g	OCH_3_	H	2g^28^	MeOH	69
3h	OCH_3_	OH	2h	MeOH	90
3i	OCH_3_	OCH_3_	2i	MeOH	73
3J	OCH_3_	OBn	2j	MeOH	67
3k	OBn	H	2k^27^	MeOH	85
3l	OBn	OCH_3_	2l^27^	MeOH	68
3m	OBn	OBn	2m^27^	MeOH	74

### General procedure of synthesis of flavonols (3d–f)

The flavonols 3k–m (3 mmol) was dissolved in THF (40 mL) and 300 mg of Pd/C 10% was added. The mixture was stirred for 12 h at room temperature under hydrogen atmosphere. The Pd/C was filtered off. After the solvent was evaporated, the precipitate washed with MeOH (10 mL) and filtered. The resulting solid was concentrated under vacuum to obtain a pure product.

#### 4′-Hydroxyflavonol (3-hydroxy-2-(4-hydroxyphenyl)-4*H*-chromen-4-one) (3a)^29^

^1^H NMR (500 MHz, DMSO-d6), *δ*, ppm: 10.08 (s, 1H), 9.33 (s, 1H), 8.10 (t, *J* = 7.7 Hz, 3H), 7.79–7.72 (m, 2H), 7.44 (s, 1H), 6.93 (d, *J* = 8.3 Hz, 2H). 13C NMR (126 MHz, DMSO-*d*_6_), *δ*, ppm: 172.5, 159.1, 154.4, 146.1, 137.8, 133.3, 129.5, 124.7, 124.4, 122.0, 121.3, 118.2, 115.4 (SI Fig. S1). Mass spectrum, *m*/*z* (*I*_rel_, %): 253.0 [M − H]-(100) (SI Fig. S14).

#### 4′-Methoxyflavonol (3-hydroxy-2-(4-methoxyphenyl)-4*H*-chromen-4-one) (3b)^29^

^1^H NMR (500 MHz, DMSO-d6), *δ*, ppm: 9.46 (s, 1H), 8.20 (d, *J* = 8.6 Hz, 2H), 8.10 (d, *J* = 8.0 Hz, 1H), 7.82–7.72 (m, 2H), 7.45 (t, *J* = 7.4 Hz, 1H), 7.13 (d, *J* = 8.6 Hz, 2H), 3.84 (s, 3H). 13C NMR (126 MHz, DMSO-*d*_6_), *δ*, ppm: 172.6, 160.4, 154.4, 145.5, 138.1, 133.4, 129.4, 124.7, 124.4, 123.5, 121.3, 118.3, 114.0, 55.3 (Fig. S2). Mass spectrum, *m*/*z* (*I*_rel_, %): 157.0 (100), 269.0 [M + 1]^+^ (55) (Fig. S15).

#### 4′-Benzoxyflavonol (2-(4-(benzyloxy)phenyl)-3-hydroxy-4*H*-chromen-4-one) (3c)^26^

^1^H NMR (500 MHz, DMSO-d6), *δ*, ppm: 9.31 (s, 1H), 8.18 (d, 2H), 8.09 (d, *J* = 8.2 Hz, 1H), 7.79–7.68 (m, 2H), 7.49–7.29 (m, 6H), 7.18 (d, *J* = 8.0 Hz, 2H), 5.18 (d, *J* = 2.5 Hz, 2H). 13C NMR (126 MHz, DMSO-*d*_6_), *δ*, ppm: 172.6, 159.5, 154.4, 145.5, 138.2, 136.6, 133.4, 129.3, 128.4, 127.9, 127.7, 124.7, 124.4, 123.8, 121.3, 118.3, 114.8, 69.4 (Fig. S3). Mass spectrum, *m*/*z* (*I*_rel_, %): 345.2 [M + 1]^+^ (92), 367.2(8) (Fig. S16).

#### 3′-Hydroxyflavonol (3-hydroxy-2-(3-hydroxy-4-methoxy-phenyl)-4*H*-chromen-4-one) (3d)^29^

^1^H NMR (400 MHz, DMSO-*d*_6_), *δ*, ppm: 9.29–9.21 (m, 2H), 8.11 (dd, *J* = 8.0, 1.6 Hz, 1H), 7.82–7.67 (m, 4H), 7.45 (t, *J* = 7.4 Hz, 1H), 7.10 (d, *J* = 8.6 Hz, 1H), 3.86 (s, 3H). 13C NMR (101 MHz, DMSO-*d*_6_), *δ*, ppm: 172.5, 154.4, 149.3, 146.2, 145.6, 138.2, 133.4, 124.7, 124.4, 123.7, 121.3, 119.7, 118.2, 114.8, 111.8, 55.6 (Fig. S4). Mass spectrum, *m*/*z* (*I*_rel_, %): 157.0 (100), 239.2 (23), 255.0 [M + 1]^+^ (55) (Fig. S17).

#### 3′,4′-Dihydroxyflavonol (2-(3,4-dihydroxyphenyl)-3-hydroxy-4*H*-chromen-4-one) (3e)^30^

^1^H NMR (400 MHz, DMSO-d6), *δ*, ppm: 9.39 (s, 3H), 8.09 (dd, *J* = 7.9, 1.7 Hz, 1H), 7.80–7.66 (m, 3H), 7.61 (dd, *J* = 8.4, 2.2 Hz, 1H), 7.43 (t, *J* = 7.4 Hz, 1H), 6.91 (d, *J* = 8.4 Hz, 1H). ^13^C NMR (101 MHz, DMSO-*d*_6_), *δ*, ppm: 172.9, 154.8, 148.1, 146.6, 145.6, 138.4, 133.8, 125.2, 124.8, 122.8, 121.8, 120.5, 118.6, 116.1, 115.8 (Fig. S5). Mass spectrum, *m*/*z* (*I*_rel_, %): 271.0 [M + 1]^+^ (88), 272.0 (8) (Fig. S18).

#### 3′-Hydroxy-4′-methoxyflavonol (3-hydroxy-2-(3-hydroxy-4-methoxyphenyl)-4*H*-chromen-4-one) (3f)^31^

^1^H NMR (400 MHz, DMSO-*d*_6_), *δ*, ppm: 9.29–9.21 (m, 2H), 8.11 (dd, *J* = 8.0, 1.6 Hz, 1H), 7.82–7.67 (m, 4H), 7.45 (t, *J* = 7.4 Hz, 1H), 7.10 (d, *J* = 8.6 Hz, 1H), 3.86 (s, 3H). ^13^C NMR (101 MHz, DMSO-*d*_6_), *δ*, ppm: 172.5, 154.4, 149.3, 146.2, 145.6, 138.2, 133.4, 124.6, 124.4, 123.7, 121.3, 119.7, 118.2, 114.8, 111.8, 55.6 (Fig. S6). Mass spectrum, *m*/*z* (*I*_rel_, %): 285.2 [M + 1]^+^ (94), 286.2 (6) Fig. S19).

#### 3′-Methoxyflavonol (3-hydroxy-2-(3-methoxyphenyl)-4*H*-chromen-4-one) (3g)^29^

^1^H NMR (400 MHz, DMSO-*d*_6_), *δ*, ppm: 9.66 (s, 1H), 8.12 (d, *J* = 7.6 Hz, 1H), 7.86–7.75 (m, 5H), 7.53–7.43 (m, 3H), 7.10 (dd, *J* = 8.2, 2.6 Hz, 1H), 3.83 (s, 4H). ^13^C NMR (126 MHz, DMSO-d6), *δ*, ppm: 173.5, 159.7, 155.0, 145.3, 139.7, 134.3, 133.0, 130.2, 125.3, 125.1, 121.7, 120.5, 119.0, 115.7, 113.9, 55.7 (Fig. S7). Mass spectrum, *m*/*z* (*I*_rel_, %): 269.0 [M + 1]^+^ (93), 270.0 (7) (Fig. S20).

#### 4′-Hydroxy-3′-methoxyflavonol (3-hydroxy-2-(4-hydroxy-3-methoxyphenyl)-4*H*-chromen -4-one) (3h)^32^

^1^H NMR (500 MHz, DMSO-*d*_6_), *δ*, ppm: 9.55 (s, 2H), 8.09 (d, *J* = 7.9 Hz, 1H), 7.83 (s, 1H), 7.80–7.73 (m, 3H), 7.50–7.40 (m, 1H), 6.96 (dd, *J* = 8.5, 3.7 Hz, 1H), 3.86 (s, 3H). 13C NMR (126 MHz, DMSO-*d*_6_), *δ*, ppm: 172.5, 154.3, 148.7, 147.4, 145.9, 138.0, 133.3, 124.6, 124.4, 122.2, 121.8, 121.2, 118.3, 115.5, 111.7, 55.7 (Fig. S8). Mass spectrum, *m*/*z* (I_rel_, %): 285.0 [M + 1]^+^ (96), 285.0(3) (Fig. S21).

#### 3′,4′-Dimethoxyflavonol (2-(3,4-dimethoxyphenyl)-3-hydroxy-4*H*-chromen-4-one) (3i)^33^

^1^H NMR (500 MHz, DMSO-*d*_6_), *δ*, ppm: 9.47 (s, 1H), 8.09 (d, *J* = 7.9 Hz, 1H), 7.86 (d, *J* = 8.6 Hz, 1H), 7.83–7.75 (m, 3H), 7.48–7.42 (m, 1H), 7.14 (d, *J* = 8.6 Hz, 1H), 3.84 (s, 6H). ^13^C NMR (151 MHz, DMSO-*d*_6_), *δ*, ppm: 173.1, 154.9, 150.8, 148.9, 145.9, 138.7, 133.9, 125.2, 124.9, 124.1, 122.0, 121.7, 118.9, 112.0, 111.5, 56.1, 56.1 (Fig. S9). Mass spectrum, *m*/*z* (*I*_rel_, %): 299.2 [M + 1]^+^ (91), 300.2 (15) (Fig. S22).

#### 3′-Methoxy-4′-benzoxyflavonol(2-(4-(benzyloxy)-3-methoxyphenyl)-3-hydroxy-4*H*-chromen-4-one) (3j)^34^

^1^H NMR (500 MHz, DMSO-*d*_6_), *δ*, ppm: 9.48 (s, 1H), 8.09 (d, *J* = 7.3 Hz, 1H), 7.86–7.72 (m, 4H), 7.50–7.30 (m, 6H), 7.22 (d, *J* = 8.8 Hz, 1H), 5.17 (s, 2H), 3.85 (s, 3H). ^13^C NMR (126 MHz, DMSO-*d*_6_), *δ*, ppm: 172.6, 154.4, 149.3, 148.7, 145.3, 138.3, 136.7, 133.4, 128.4, 127.9, 127.9, 124.7, 124.4, 123.9, 121.3, 121.2, 118.3, 113.0, 111.3, 69.8, 55.7 (Fig. S10). Mass spectrum, *m*/*z* (*I*_rel_, %): 375.2 [M + 1]^+^ (97), 397.2 (3) (Fig. S23).

#### 3′-Benzoxyflavonol (2-(3-(benzyloxy)phenyl)-3-hydroxy-4*H*-chromen-4-one) (3k)

^1^H NMR (400 MHz, DMSO-*d*_6_), *δ*, ppm: 9.69 (s, 1H), 8.11 (d, *J* = 8.0 Hz, 1H), 7.89–7.71 (m, 4H), 7.53–7.29 (m, 7H), 7.15 (dd, *J* = 8.4, 2.7 Hz, 1H), 5.17 (s, 2H). ^13^C NMR (126 MHz, DMSO-*d*_6_), *δ*, ppm: 173.0, 158.2, 154.5, 144.7, 139.2, 136.8, 133.7, 132.5, 129.6, 128.4, 127.9, 127.8, 124.7, 124.5, 121.2, 120.2, 118.4, 115.9, 114.3, 69.4 (Fig. S11). White solid with a yellowish tint, mp 137–138 °C. Mass spectrum, *m*/*z* (*I*_rel_, %): [M + H]^+^ = 345.0 [M + 1]^+^ (97), 346 (20) (Fig. S24).

#### 3′-Benzoxy-4′-methoxyflavonol (2-(3-(benzyloxy)-4-methoxyphenyl)-3-hydroxy-4*H*-chromen-4-one) (3l)^31^

^1^H NMR (400 MHz, DMSO-*d*_6_), *δ*, ppm: 9.50 (s, 1H), 8.10 (dd, *J* = 8.0, 1.6 Hz, 1H), 7.94–7.87 (m, 2H), 7.84–7.72 (m, 2H), 7.55–7.30 (m, 6H), 7.21–7.14 (m, 1H), 5.18 (s, 2H), 3.86 (s, 3H). ^13^C NMR (126 MHz, DMSO-d6), *δ*, ppm: 172.6, 154.3, 150.6, 147.4, 145.3, 138.3, 136.9, 133.4, 128.4, 128.1, 127.9, 124.7, 124.5, 123.5, 121.9, 121.2, 118.3, 112.7, 111.7, 70.2, 55.6 (Fig. S12). Mass spectrum, *m*/*z* (*I*_rel_, %): 375.0 [M + 1]^+^ (88), 376.0(12) (Fig. S25).

#### 3′,4′-Dibenzoxyflavonol (2-(3,4-bis(benzyloxy)phenyl)-3-hydroxy-4*H*-chromen-4-one) (3m)^26^

^1^H NMR (500 MHz, DMSO-d6), *δ*, ppm: 9.52 (s, 1H), 8.09 (d, *J* = 8.0 Hz, 1H), 7.94 (s, 1H), 7.87 (d, *J* = 8.6 Hz, 1H), 7.83–7.70 (m, 2H), 7.54–7.28 (m, 11H), 7.25 (d, *J* = 8.7 Hz, 1H), 5.22 (d, *J* = 7.3 Hz, 4H). ^13^C NMR (126 MHz, DMSO-d6), *δ*, ppm: 172.6, 154.3, 149.8, 147.7, 145.2, 138.4, 137.0, 136.8, 133.4, 128.4, 127.9, 127.8, 127.6, 124.7, 124.4, 123.9, 121.9, 121.2, 118.3, 113.7, 70.4, 69.9 (Fig. S13). Mass spectrum, *m*/*z* (*I*_rel_, %): 451.0 [M + 1]^+^ (74), 452.0 (16) (Fig. S26).

### Quantum-chemical calculations

Unconstrained and constrained geometry optimizations of isolated molecules of flavonols in the ground electronic state and analysis of rotation barriers were carried out at the DFT levels of theory using the B3LYP functional^35^ and cc-pVDZ basis set^36^ implemented in the GAUSSIAN 16 program package.^37^ The solvent (dichloromethane and acetonitrile) effect at 298.14 K and standard pressure was estimated at the level of the Polarized Continuum Model (PCM).^38^

### Spectroscopic measurements

#### NMR spectra

^1^H NMR spectra were recorded using Unity Inova 400, Bruker Avance DRX 500, and Bruker Avance III 400 MHz spectrometers in DMSO-*d*_6_. 13C NMR spectra were recorded on Bruker Avance DRX 500 and Agilent ProPulse 500 MHz spectrometers at a resonance frequency of 126 MHz in DMSO-*d*_6_. Chemical shifts are reported in the δ scale (ppm). Mass spectra were recorded on an Agilent 1100 high-performance liquid chromatograph (HPLC) equipped with a diode matrix and an Agilent LC/MSD SL mass-selective detector, a SUPELCO Ascentis Express C18 chromatographic column 2.7 μm 4.6 mm × 15 cm. Control over the course of the reaction and the individuality of the obtained substances was carried out by TLC method on silica gel-coated “Polychrom SI F254” plates with a fluorescent detector in the hexane–ethyl acetate 2 : 1 system, the developer was an ultraviolet lamp. If necessary, additional purification of the obtained compounds was carried out using flash chromatography (UPFP) on a PuriFlash XS520 Plus device using gradient elution. The melting points of all synthesized compounds were determined using a Hanon Instruments MP450 open capillary tube automatic melting point apparatus.

#### UV-vis and fluorescence spectra

Absorption and fluorescence spectra of flavonols in liquid media were registered in dichloromethane and acetonitrile solutions. Commercial solvents were additionally rectified and dried before use. The flavonols' concentrations in investigated solutions ranged from 1.0 × 10–5 to 5.0 × 10–5 M l^−1^. Absorption and fluorescence spectra were recorded on a PerkinElmer LS55 spectrofluorimeter and a Shimadzu UV-2401PC spectrophotometer. The latter was also used to record the excitation and fluorescence spectra of flavonols in the solid state.

#### Fluorescence titrations

The interaction between flavonols and protein molecules was studied using β-glucosidase (β-D-glucoside glucohydrolase) from Almonds purchased from Sigma-Aldrich as a lyophilized powder with > 98% purity. Stock solutions of flavonols were prepared by dissolving their samples in DMSO. A stock solution of β-glucosidase was prepared by dissolving a sample of the enzyme in an aqueous phosphate buffer with pH 6.86. The fluorescence was excited at 380 nm and the emission spectra were recorded in the range of 400–700 nm using a Hitachi 850 spectrofluorimeter. The experimental details are described elsewhere.^39^

#### X-ray diffraction study

X-Ray diffraction studies were performed on an automatic “Bruker APEX II” diffractometer (graphite monochromated MoKα radiation, CCD-detector, φ- and ω-scanning). The structures were solved by direct method using OLEX2 ref. 40 package with SHELXT^41^ and SHELXL modules.^42^ The phenyl ring of the benzyl group in structure 3k is disordered over two positions (A and B) due to rotation around the C_sp^3^_–C_ar_ bond. The restrictions on the bond lengths in the disordered fragment were applied (C_ar_–C_ar_ = 1.39 Å). The hydrogen atoms were placed in their ideal positions and refined by “riding” model with *U*_iso_ = n*U*_eq_ of the carrier atom (*n* = 1.5 for methyl groups and *n* = 1.2 for other hydrogen atoms). The crystallographic data and experimental parameters are listed in Table S1 in Supplementary section. Deposition Numbers CCDC 2416411 for structure 3b and CCDC 2416347 for structure 3k contain the supplementary crystallographic data for this paper. These data can be obtained free of charge by the joint Cambridge Crystallographic Data Centre and Fachinformationszentrum Karlsruhe Access Structures service http://www.ccdc.cam.ac.uk/structures.

### Molecular docking setup

The molecular docking setting up was carried out with the AutoDock Tools (ADT) software, version 1.5.7.^43^ The addition of hydrogen, the calculation of the Gasteiger charges of the receptor, and ligands were also performed using the ADT software. Molecular docking calculations were performed with the AutoDock Vina 1.1.2 software.^43,44^ The 3D X-ray structure of *Paenibacillus polymyxa* β-glucosidase B (BglB, PDB 2O9R),^45^*Raucaffricine* β-glucosidase (rBG, PDB 4A3Y),^46^*Thermotoga maritima* β-glucosidase (TmGH1, PDB 1OD0),^47^ and human cytosolic β-glucosidase (hCBG, PDB 2JFE)^48^ were downloaded from the RCSB Protein Data Bank. The calculations details were described in details elsewhere.^26,39^

## Results and discussion

### Absorption and fluorescence spectra in solutions

Research into the electronic transitions of flavonol molecules, conducted using both semi-empirical methods and density functional theory (DFT), has revealed that broad absorption bands in the 340–380 nm and 240–270 nm ranges result from intense electronic transitions occurring primarily between molecular orbitals located on the chromone fragment of these molecules.^49^ In this case, the side benzene ring can affect the electron density distribution within the chromone fragment, which may result in a shift in the absorption bands. Therefore, according to chromophore theory, the side benzene ring operates as an auxochrome of a complex structure. Electronic transitions involving inter-fragmental charge transfer between the side benzene ring and the chromone part of the molecule typically occur in the short-wavelength region of the spectrum. However, when a strong electron donor, such as –N(CH_3_)_2_, or a strong electron acceptor, such as –NO_2_, is present in the side ring, the electronic transition shifts to the long-wavelength region. This transition can occur either from the molecular orbitals localized on the benzene ring to the orbitals of the chromone fragment, or *vice versa*. The presence of a charge transfer transition is manifested by the appearance of a new long-wavelength absorption band in the range of 400–430 nm.^12^Table 2 summarizes the calculated absorption and fluorescence parameters of flavonols 3a–f in water.

Calculated absorption and fluorescence parameters of flavonols 3a–f in water^a^

**Table:** 

Transitions	I (S_0_ → S_1_)					II (S_0_ → S_2_)			
Flavonol	*λ*_abs_, nm (*ν*_abs_, cm^−1^)	*f*	C.I.	Type	ICT	*λ*_abs_, nm (*ν*_abs_, cm^−1^)	*f*	C.I.	Type
H	349 (28 625)	0.354	0.69*χ*_1→1’_	CHR	0.102	308 (32 520)	0.000	0.70*χ*_4→1’_	n → π*
3′-OH	356 (28 110)	0.308	0.68*χ*_1→1’_	CHR	0.133	332 (30 120)	0.041	0.68*χ*_2→1’_	CT
4′-OH	361 (27 695)	0.435	0.70*χ*_1→1’_	CHR	0.152	306 (32 730)	0.000	0.70*χ*_4→1’_	n → π*
3′,4′-OH	372 (26 855)	0.421	0.70*χ*_1→1’_	CHR	0.232	315 (31 750)	0.068	0.70*χ*_2→1’_	FL

a *λ*_abs_, *ν*_abs_ – wavelength and wave number of the electronic transition, *f* – transition oscillator strength, C.I. – configuration composition of the transition, types of transitions – CHR – local, located predominantly on the chromone fragment, CT – transition with interfragment charge transfer, FL – transition localized on the molecule as a whole, *n* → π* – forbidden nπ* transition. ICT – value of interfragment charge transfer during the S_0_ → S_1_ transition.

**Table:** 

Transitions	III (S_0_ → S_3_)				IV (S_0_ → S_4_)			
	*λ*_abs_, nm (*ν*_abs_, cm^−1^)	*f*	C.I.	Type	*λ*_abs_, nm (*ν*_abs_, cm^−1^)	*f*	C.I.	Type
H	292 (34 190)	0.116	0.70*χ*_2→1’_	FL	284 (35 195)	0.137	0.64*χ*_3→1’_	CT
3′-OH	306 (32 680)	0.000	0.70*χ*_4→1’_	n→π*	289 (34 600)	0.137	0.64*χ*_3→1’_	FL
4′-OH	298 (33 595)	0.089	0.67*χ*_2→1’_	FL	282 (35 385)	0.005	0.41*χ*_1→2’_ + 0.41*χ*_1→3’_	FL
3′,4′-OH	305 (32 840)	0.000	0.70*χ*_4→1’_	n→π*	290 (34 455)	0.058	0.65*χ*_3→1’_	CT

**Fig. 1:**
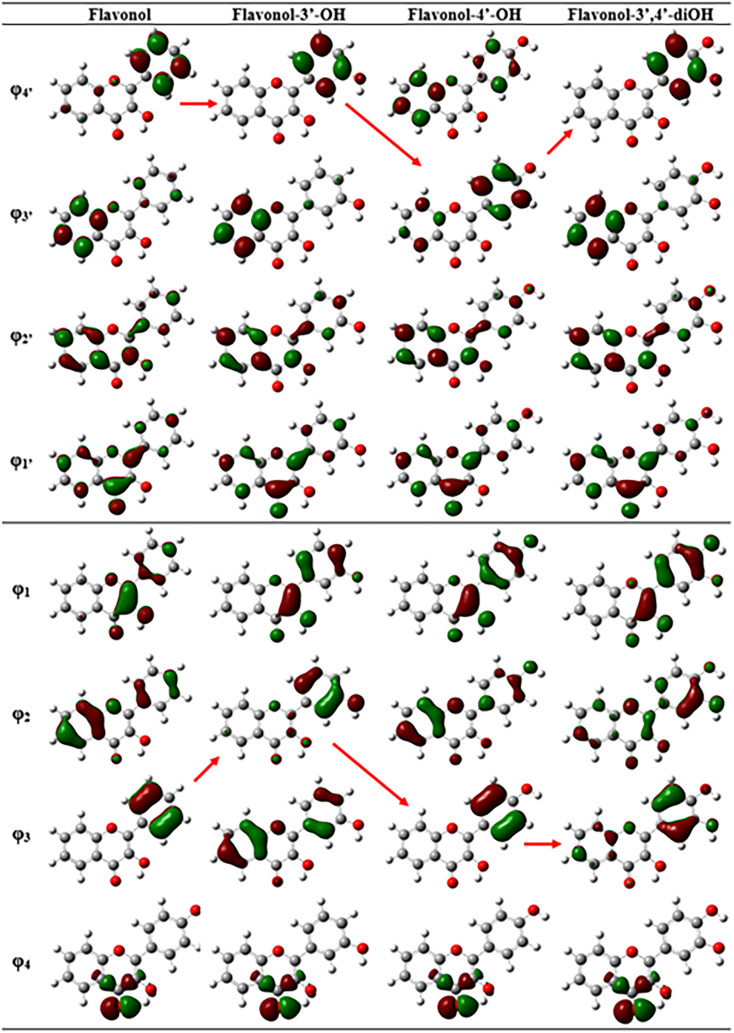
Frontier molecular orbitals of flavonol, and substituted flavonol with 3′-OH, 4′-OH, and 3′,4′-OH.

**Fig. 2:**
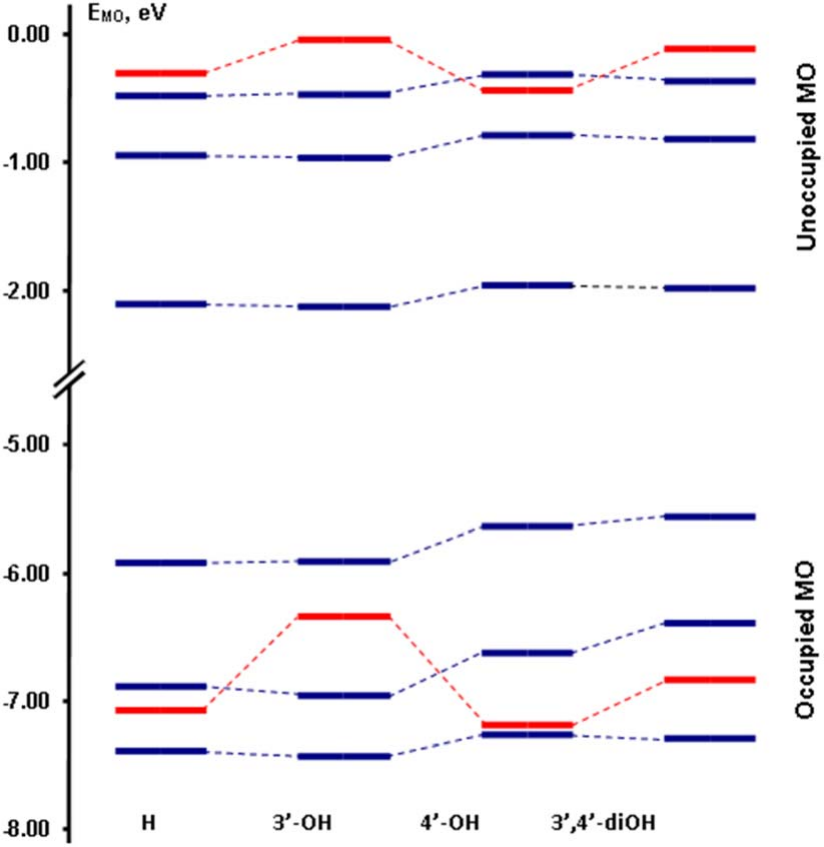
Influence of substituents' positions on MO energies in flavonols obtained using the b3lyp/cc-pVDZ level of theory (see Table 2 for more details).

The molecular orbitals of unsubstituted flavonol, as well as its monosubstituted derivatives that have hydroxyl groups in the C3′ and C4′ positions, are illustrated in Fig. 1. Meanwhile, the disubstituted derivative, 3′,4′-dihydroxyflavonol, is also included in this analysis. Fig. 2 presents a diagram showing the relative energies of these molecular orbitals. Additionally, Table 2 provides details on the theoretical parameters of long-wavelength transitions observed in the spectra of these compounds. The analysis of configurational interaction reveals that the long-wavelength intense transitions of all flavonols can be characterized by a transition between the highest occupied molecular orbital (MO) *φ*_1_ and the lowest vacant molecular orbital *φ*_1_′ The MO *φ*_1_ is delocalized between the chromone and the side phenyl fragment. However, as the torsion angle between these fragments increases, the localization of MO *φ*_1_ increasingly favors the chromone fragment. In contrast, the vacant MO *φ*_1_' is primarily localized on the chromone fragment. Therefore, it can be concluded that the long-wavelength electron transition generally exhibits a “chromone” nature (CHR).

This conclusion is supported by the relatively low values of interfragment charge transfer during the transition from the ground state to the Frank–Condon excited state S_1_, which are approximately 0.1–0.2 electronic charges (e^−^) only. These low values suggest that the side phenyl ring does not significantly participate in the excitation process.

The Table 2 illustrates that increasing the number of substituents in the phenyl side ring results in a decrease in S_0_ → S_1_ transition energy, *i.e.* to a bathochromic shift of the corresponding absorption band from 350 nm to 370 nm approximately. Adding a substituent at position C4′ enhances the transition oscillator strength *f*, which in turn increases the intensity of the associated absorption band. Conversely, introducing a substituent at position C3′ has the opposite effect, reducing the intensity of the absorption band.

DFT calculations of the electronic spectrum for the compound featuring an OH group at the C3′ position reveal an additional long-wavelength band in the ∼330 nm range. This band results from an electronic transition between the *φ*_2_ orbital, which is localized on the side phenyl ring, and the *φ*_1_′ orbital, which is localized on the chromone fragment. This transition is associated with significant inter-fragment charge transfer (ICT). In the spectra of other compounds, similar transitions are observed in the shorter wavelength region of 285–290 nm (see Table 2). This phenomenon occurs due to an increase in the energy of the corresponding occupied molecular orbital when a substituent is introduced at the C3′ position. In Fig. 2, the energies of the molecular orbitals localized on the side phenyl ring are highlighted in red. In the 305–310 nm spectrum range, all compounds have a forbidden *n*–π* transition from the n-orbital *φ*_4_ to the orbital φ_1_′. The short-wave intense band ranging from 280 to 315 nm in all spectra is attributed to the electronic transition *φ*_2_ → *φ*_1_′ (with *φ*_3_ → *φ*_1_′ occurring in the compound substituted at the C3′ position). As illustrated in Fig. 1, the *φ*_2_ orbital is delocalized across both fragments of the flavonol molecule. Therefore, the transition from *φ*_2_ to *φ*_1_′ is not localized and can be understood as the excitation of the molecule as a whole. It should be noted that the results from the DFT method correlate well with semi-empirical methods.^49^

**Fig. 3:**
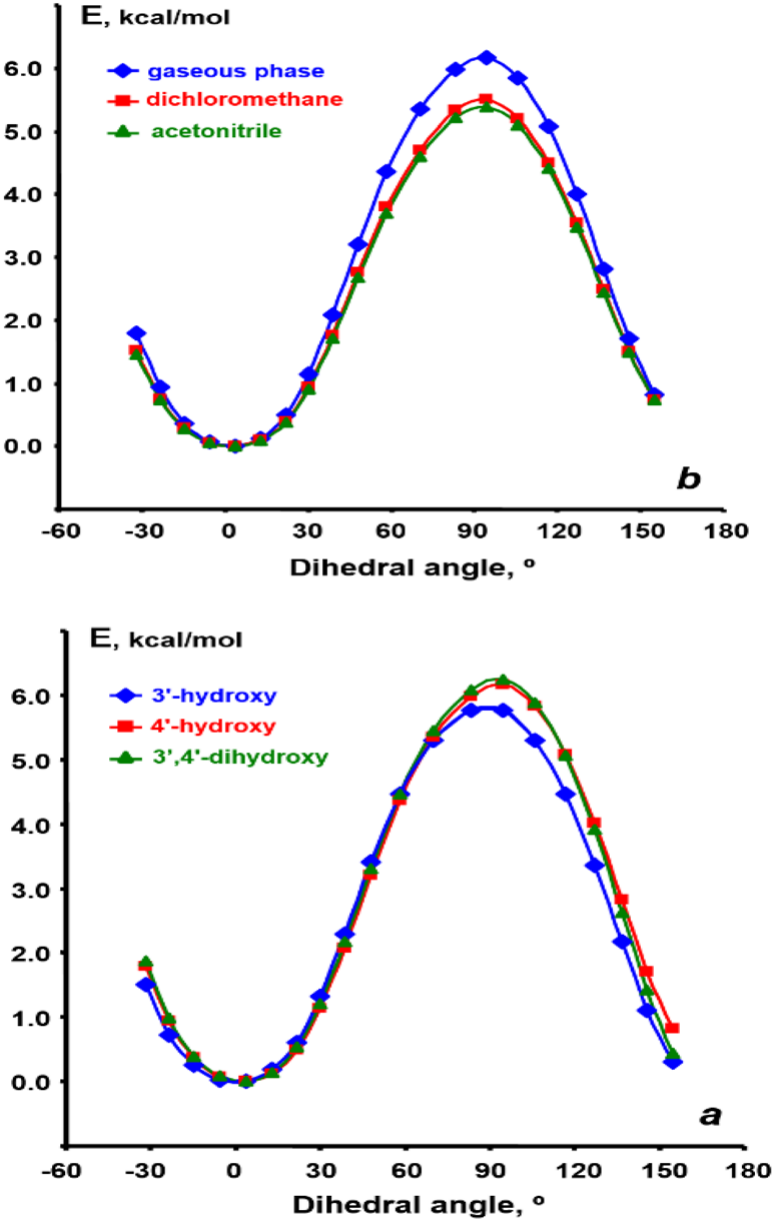
DFT-calculated rotation barriers between the chromone moiety and phenyl side ring calculated using b3lyp/cc-pVDZ level of theory. (a) – if substituents are in different positions of the benzene ring, (b) – for the same flavonol 3a in different media.

The impact of the side phenyl ring on the chromone fragment arises from both the electronic effects of its substituents and the extent of their electronic conjugation governed by the torsion angle between the planes of the both fragments. The conformations of flavonol molecules in the gas phase, as well as in dichloromethane and acetonitrile, were investigated by modeling the potential energy curves associated with the rotation of the side ring relative to the plane of the chromone fragment. This rotation was analyzed over an angle range from −30° to 150° for 3′-hydroxy-, 4′-hydroxy-, and 3′,4′-dihydroxyflavonols. As illustrated in Fig. 3, regardless of the substituent's position, the minimum potential energy occurs at angles between −10° and 10°. This observation indicates that over 97% of flavonol molecules are planar in both solutions and the gas phase. Furthermore, the calculations reveal that an increase in the polarity of the medium results in a decrease in the rotation barrier by approximately 0.6 to 0.7 kcal mol^−1^.

The influence of electronic effects from substituents is quantified by the Hammett constants *σ*_para_ and *σ*_meta_. However, since multiple substituents can simultaneously occupy the para and meta positions in the side ring, and considering the dampening effect of the aromatic π-system, we calculated the overall *σ*_para_ constants for various RO-phenyl substituents using the web tool outlined in the article.^51^ The calculated *σ*_para_ values are presented in Table 3. It is important to note that these *σ*_para_ values are notably low, indicating a very weak influence of the substituents in the side benzene ring on the electronic structure of the chromone moiety of the molecules.

**Table 3:** Spectral characteristics of long-wavelength absorption bands of the flavonols in dichloromethane and acetonitrile^a^

Flavonol	3′-R	4′-R	*σ*_para_	Dichloromethane		Acetonitrile		Δ*ν*_abs_
				*λ*_abs_	*ν*_abs_	*λ*_abs_	*ν*_abs_	
3^b^	H	H	0.000	344	29 070	340	29 400	330
3a	H	OH	−0.047	351	28 490	350	28 570	80
3b	H	OCH_3_	−0.037	352	28 410	350	28 570	160
3c	H	OBn	−0.007	355	28 170	349	28 655	485
3d	OH	H	+0.007	346	28 900	343	29 155	255
3e	OH	OH	−0.046	353	28 330	355	28 170	−160
3f	OH	OCH_3_	−0.040	356	28 090	354	28 250	160
3g	OCH_3_	H	+0.017	345	28 984	342	29 240	256
3h	OCH_3_	OH	−0.041	357	28 010	355	28 170	160
3i	OCH_3_	OCH_3_	−0.037	361	27 700	356	28 090	390
3j	OCH_3_	OBn	−0.003	361	27 700	356	28 090	390
3k	OBn	H	+0.032	346	28 900	343	29 155	255
3l	OBn	OCH_3_	−0.005	360	27 780	356	28 090	310
3m	OBn	OBn	−0.001	361	27 700	355	28 170	470

a *σ*_para_ – Hammett constants for R-oxyphenyl fragments, *λ*_abs_ – absorption band maxima in nm scale, *ν*_abs_ – absorption band maxima in cm^−1^ scale, Δ*ν*_abs_– shifts of absorption band maxima on going from dichloromethane to acetonitrile.

b Spectral data for unsubstituted flavonol 3 were taken from ref. 50 The data for nonpolar medium are shown for chloroform.

A comparison of the *σ*_para_ constants with the positions of the long-wavelength absorption bands in the experimental spectra of flavonols revealed no correlation between them.

**Fig. 4:**
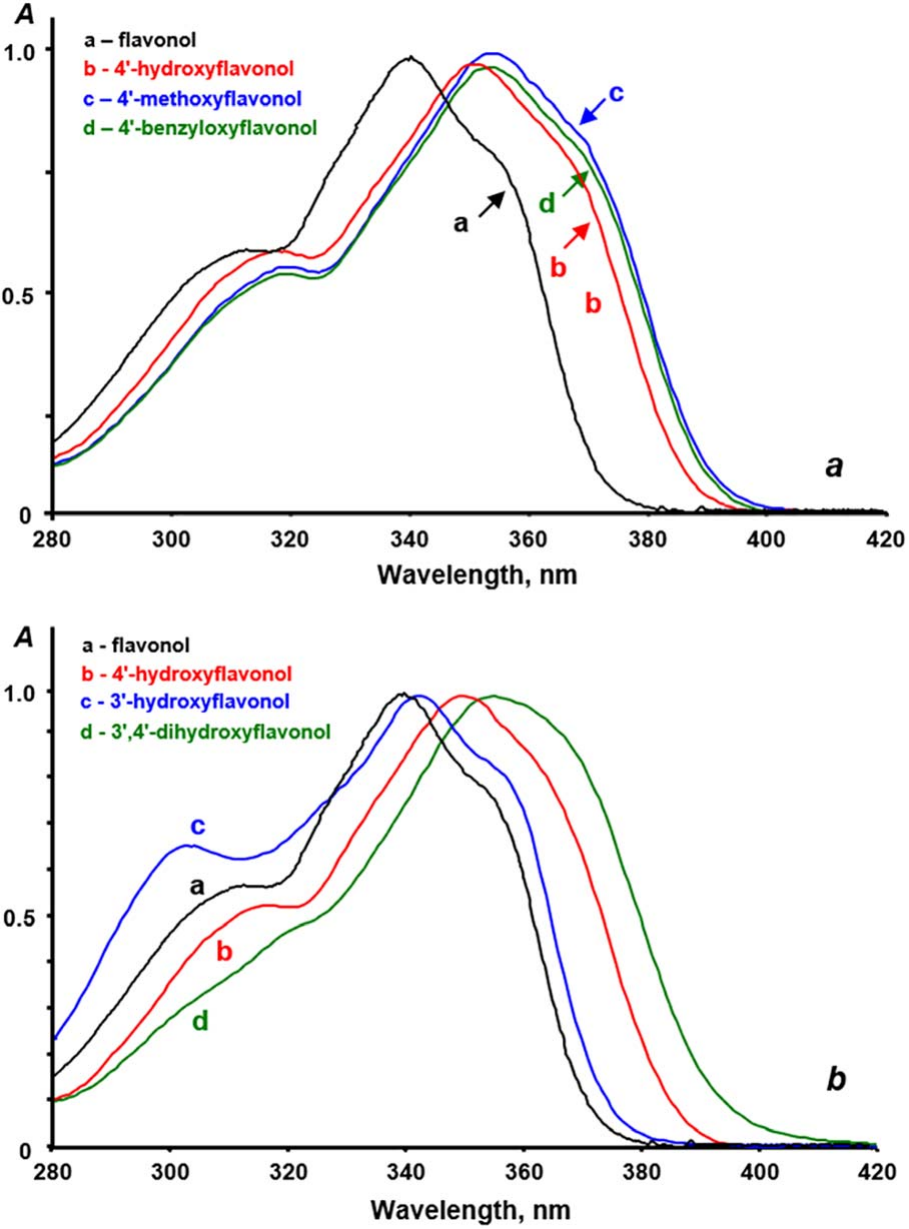
Normalized absorption spectra of the studied flavonols in dichloromethane. a – different substituents in the same position of the side benzene ring, b – the same substituents in different positions of the side benzene ring. Concentrations of flavonols were in a range of 1–5·10^−5^ M.

This indicates that substituents do not have a significant impact on the spectral parameters of flavonols. For example, substituting a hydroxy group with either a methoxy group or a benzyloxy group in the same position on the aromatic ring does not cause any shift in the absorption band (see Table 3 and Fig. 4a). Additionally, no shifts in the band position were observed with any combinations of substituents in 3′,4′-disubstituted flavonols.

More significant differences in the absorption band parameters are observed depending on the positions of the substituents in the benzene ring (see Fig. 4b). For 3′-R-hydroxyflavonols in dichloromethane, regardless of the type of substituents, the absorption maximum falls within the range of 345–346 nm (28 900–28985 cm^−1^). The *σ*_para_ constants for these compounds have positive values of +0.01 to +0.03, indicating a very weak electron-withdrawing effect of the benzene ring on the chromone fragment. In contrast, 4′-R-oxyflavonols and 3′,4′-di-R-hydroxyflavonols exhibit long-wavelength band maxima in approximately similar range: at 351–355 nm (28 170–28490 cm^−1^) and 353–361 nm (27 700–28330 cm^−1^), respectively. The corresponding *σ*_para_ constants for these compounds range from −0.01 to −0.05, suggesting that the benzene ring demonstrates weakly expressed electron-releasing properties. When going to a more polar environment, such as acetonitrile, a slight hypsochromic shift of the absorption bands from 80 to 500 cm^−1^ is observed (see Table 3). Additionally, the effect of substituent position is even less pronounced in acetonitrile.

The *σ*_para_ values indicate that substituents on the side benzene ring have a very weak effect on the spectral properties of flavonols. In contrast, the introduction of 4′-diethylamino- and 4′-nitro substituents into the side ring results in significant changes to the absorption spectra. In these latter cases, the *σ*_para_ values are tens of times greater, measuring −0.146 and +0.372, respectively. Natural flavonols commonly contain hydroxy and various alkoxy groups, which typically have low *σ*_para_ values. Therefore, it can be assumed that natural flavonols with a similar structure in the chromone moiety of their molecules will exhibit approximately the same absorption spectra, regardless of the type or location of substituents in the side phenolic ring.

Due to the ESIPT process, the fluorescence spectra of flavonols can exhibit two distinct fluorescence bands. The first is a short-wavelength emission band corresponding to the excited form of the original flavonol (N*), and the second is a long-wavelength emission band from the phototautomer (T*), which is formed by the transfer of a proton from the 3-hydroxy group to the carbonyl group of the chromone fragment. Proton transfer is typically a very rapid and irreversible process, resulting in the fluorescence spectrum usually displaying only one long-wavelength band of the phototautomer (T*). However, as the polarity of the medium increases, the activation barrier for the proton transfer reaction also increases. Consequently, a band corresponding to the N* form may appear in the fluorescence spectra. In proton-donating solvents or when proton-donating impurities are present, flavonols can form intermolecular hydrogen bonds. This leads to suppressing the excited-state intramolecular proton transfer (ESIPT) and results in the fluorescence of the N* form. Additionally, substituents on the side ring of flavonols can influence the dynamics of phototautomerization. Specifically, introducing an electron-releasing group at the C4′ position may decrease the acidity of the 3-hydroxy group in the chromone ring and slow down ESIPT, which can lead to the emergence of two-band fluorescence.

**Fig. 5:**
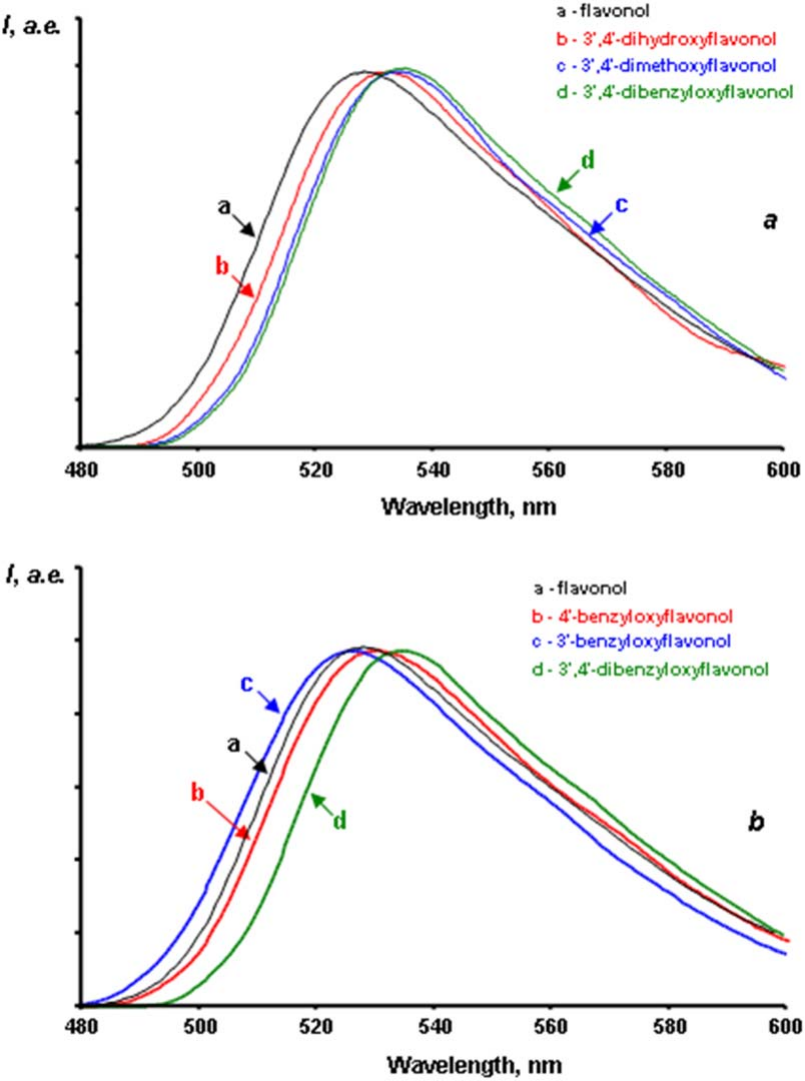
Fluorescence spectra of studied flavonols in dichloromethane. a – different substituents in the same position of the side benzene ring, b – the same substituents in different positions of the side benzene ring. Concentrations of flavonols were in a range of 1–5·10^−5^ M.

The experimental fluorescence spectra of all the studied flavonols exhibited a single emission band. This observed emission band is attributed to the T* phototautomer, as indicated by the high Stokes shift values in the range of 8900–10500 cm^−1^ (see Table 4). A trace amount of fluorescence from the N* form can be detected in the short-wavelength region of the spectra. However, this may be attributed to water impurities that were adsorbed by acetonitrile during the experiment, as well as the formation of trace amounts of flavonol hydrates, which may not undergo proton phototransfer. Considering the low *σ*_para_ values and the spectral parameters outlined in Table 3, we can conclude that the fluorescence spectra of natural flavonols in aprotic solvents will be single-band, regardless of the nature and position of the substituents in the side benzene ring. The effects of the nature and position of these substituents are illustrated in Fig. 5a and b, while the quantitative parameters of the emission bands are provided in Table 3.

**Table 4:** Spectral characteristics of long-wavelength emission bands of the flavonols in dichloromethane and acetonitrile^a^

Flavonol	3′-R	4′-R	Dichloromethane			Acetonitrile		
			*λ*_fl_	*ν*_fl_	Δ*ν*_St_	*λ*_fl_	*ν*_fl_	Δ*ν*_St_
3^b^	H	H	530	18 865	10 200	526	18 860	10 500
3a	H	OH	528	18 940	9550	547	18 280	10 290
3b	H	OCH_3_	530	18 870	9540	532	18 795	9775
3c	H	OBn	529	18 905	9265	539	18 550	10 105
3d	OH	H	527	18 975	9925	527	18 960	10 195
3e	OH	OH	529	18 905	9425	536	18 655	9515
3f	OH	OCH_3_	534	18 725	9365	538	18 585	9665
3g	OCH_3_	H	524	19 085	9900	528	18 940	10 300
3h	OCH_3_	OH	531	18 830	9180	534	18 730	9440
3i	OCH_3_	OCH_3_	533	18 760	8940	528	18 940	9150
3j	OCH_3_	OBn	534	18 725	8975	542	18 450	9640
3k	OBn	H	525	19 050	9850	536	18 655	10 500
3l	OBn	OCH_3_	534	18 725	9055	540	18 520	9570
3m	OBn	OBn	534	18 725	8975	542	18 450	9720

a *λ*_fl_, *ν*_fl_ – these designations are given in Table 2, Δ*ν*_St_ – Stokes shifts of fluorescence, cm^−1^.

b Spectral data for unsubstituted flavonol were taken from ref. 50 data for nonpolar medium are shown for chloroform.

The analysis of the data in Table 3 indicates that the nature and position of the substituents in the side benzene ring have a lesser impact on the position of the T* emission band compared to the position of the absorption bands. Specifically, the average emission band maxima for 3′-R-oxy-, 4′-R-oxy-, and 3′,4′-R-dioxyflavonols are 525 ± 2 nm, 529 ± 1 nm, and 533 ± 4 nm, respectively. The shifts of the bands when changing the position of the substituent range from 135 to 145 cm^−1^ on the energy scale, which indicates that these shifts are relatively insignificant.

The energy costs involved in rearranging the geometry of molecules and their solvate shell is characterized by the Stokes shifts of fluorescence (Δ*ν*_St_). For the flavonols studied in dichloromethane, the values of Δ*ν*_St_ range from 9850 to 9925 cm^−1^ for 3′-R-hydroxyflavonols, 9265 to 9550 cm^−1^ for 4′-R-hydroxyflavonols, and 8940 to 9425 cm^−1^ for 3′,4′-R-dihydroxyflavonols. Notably, the lowest Stokes shift values are observed in flavonols that lack hydroxyl groups. Considering the similar mechanism and kinetics of excited-state intramolecular proton transfer (ESIPT), as well as the larger Δ*ν*_St_ values for hydroxyl-containing flavonols and the planar geometry of molecules in both the initial and phototautomeric forms (T*), we can conclude that the differences in Stokes shifts arise from the structural characteristics of the solvation shell in the ground state and its subsequent rearrangement during phototautomerization.

The changes in solvent polarity on going from dichloromethane to acetonitrile do not significantly affect the position of the phototautomer emission bands. The observed shifts in the bands are multidirectional, do not exceed a few nanometers, and are statistically insignificant. The Stokes shifts of fluorescence for 3′-R-hydroxyflavonols, 4′-R-hydroxyflavonols, and 3′,4′-R-dihydroxyflavonols in acetonitrile are within the ranges of 10 190–10500 cm^−1^, 9775–10290 cm^−1^, and 9150–9665 cm^−1^, respectively. Thus, Δ*ν*_St_ values of flavonols in more polar acetonitrile are correspondingly 460, 625, and 225 cm^−1^ higher than those in less polar dichloromethane. A comparison of the spectral characteristics of the absorption and fluorescence bands indicates that the increase in Δ*ν*_St_ is due to a hypsochromic shift of the absorption bands when going from dichloromethane to acetonitrile solutions. This suggests additional energy costs are associated with the relaxation of the solvate shell of flavonols upon excitation in polar media.

### Fluorescence spectra in the solid state

To investigate the spectral properties of flavonols in the solid phase, we grew crystals of the compounds we were studying and recorded their fluorescence spectra. Since recording the absorption spectra of the crystals was not possible, we obtained fluorescence excitation spectra for each compound instead. The shape of these fluorescence excitation spectra closely resembles that of the corresponding absorption spectra, enabling us to assess the light absorption characteristics of flavonols in their solid state.

Fluorescence excitation spectra were measured by assessing the luminescence intensity at 450 nm and 550 nm, corresponding to the emission regions of the N* and T* forms, respectively. The refined positions of the maxima of the long-wavelength band in the excitation spectra (*λ*_max_^ex^/*ν*_max_^ex^) were determined through double differentiation of the spectral curves. The differences in maxima positions in the spectra measured at 450 nm and 550 nm did not exceed 3 nm; therefore, averaged values of *λ*_max_^ex^/*ν*_max_^ex^ were utilized for further analysis of the obtained data. The spectral characteristics of flavonols in the solid state are presented in Table 5.

**Table 5:** Spectral characteristics of studied flavonols in the solid state^a^

Flavonol	3′-R	4′-R	Excitation spectra		Fluorescence spectra						
					Form N*			Form T*			*I*_N_/*I*_T_
			*λ*_ex_	*ν*_ex_	*λ*_N_	*ν*_N_	Δ*ν*_St,N_	*λ*_T_	*ν*_T_	Δ*ν*_St,T_	
3^b^	H	H	357	28 020	461	21 690	6330	547	18 290	9730	0.85
3a	H	OH	363	27 610	453	22 080	5530	553	18 080	9530	1.14
3b	H	OCH_3_	364	27 530	452	22 130	5400	545	18 350	9180	1.73
3c	H	OBn	363	27 580	467	21 394	6186	557	17 946	9634	1.26
3d	OH	H	363	27 590	475	21 060	6530	539	18 560	9030	1.13
3e	OH	OH	365	27 400	453	22 080	5320	539	18 560	8840	1.58
3f	OH	OCH_3_	363	27 570	460	21 720	5850	561	17 840	9730	1.11
3g	OCH_3_	H	362	27 660	450	22 210	5450	537	18 630	9030	1.76
3h	OCH_3_	OH	362	27 640	464	21 540	6100	550	18 180	9460	1.32
3i	OCH_3_	OCH_3_	364	27 460	486	20 550	6910	536	18 660	8800	1.57
3j	OCH_3_	OBn	365	24 625	456	21 950	5475	545	18 342	9083	1.62
3k	OBn	H	375	26 670	457	21 865	4805	555	18 030	8640	2.57
3l	OBn	OCH_3_	362	27 670	454	22 040	5630	542	18 450	9220	1.63
3m	OBn	OBn	357	28 020	461	21 690	6330	547	18 290	9730	0.85

a *λ*_ex_, *ν*_ex_ – positions of long-wavelength excitation bands in nm and cm^−1^; *λ*_N_, *ν*_N_, *λ*_T_, *ν*_T_– positions of emission bands of N* and T* forms in nm and cm^−1^; Δ*ν*_St_,_N_ and Δ*ν*_St_,_T_ – Stokes shifts of fluorescence of N* and T* forms in cm^−1^ calculated relatively long-wavelength excitation bands: Δ*ν*_St_,_N_ = *ν*_exc_ − *ν*_N_ and Δ*ν*_St_,_T_ = *ν*_exc_ − *ν*_T_; *I*_N_/*I*_T_ – ratio of intensities of forms N* and T*.

b Spectral data for unsubstituted flavonol were taken from ref. 50 data for nonpolar medium are shown for chloroform.

The data presented in the Table 5 indicate that the positions of the fluorescence excitation bands in the solid phase do not correspond with the positions of the absorption bands in flavonol solutions, nor do they align with the Hammett constants of the side ring. When going from the less polar solvent dichloromethane to the more polar solvent acetonitrile, the positions of absorption band maxima shift hypsochromically by an average of 415 cm^−1^. In contrast, moving to the crystalline phase results in a bathochromic shift of the bands by an average of 730 cm^−1^, which suggests a decrease in the environmental polarity. Additionally, flavonols in the crystalline state exhibit a smaller variation in the bands' positions.

**Fig. 6:**
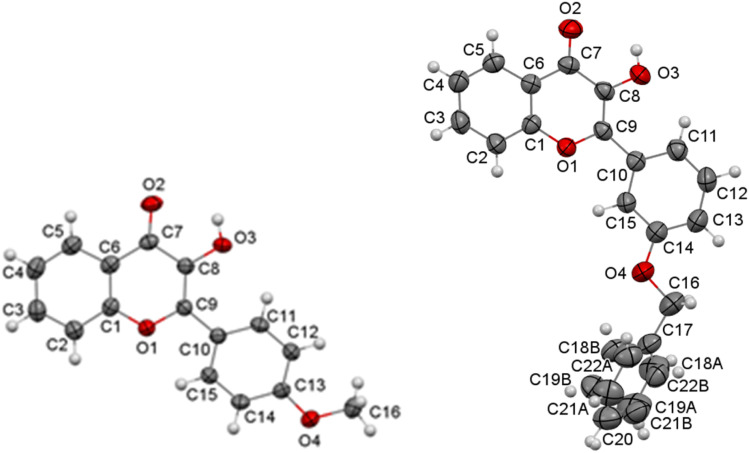
Molecular structure of compounds 3b (on the left) and 3k (on the right) according to the X-ray diffraction data. Thermal ellipsoids are shown at 50% probability level.

To understand how substituents on the side benzene ring of flavonols influence their spectral properties, X-ray structural analysis was conducted on compounds 3b and 3k. The structures and geometries of the substances studied, as obtained from X-ray diffraction, are illustrated in Fig. 6. It is important to note that the “crystallographic” numbering of atoms, assigned during structure resolution using the OLEX2 package, does not align with the atom numbering typically used for flavonols. Therefore, when referencing the “crystallographic” atom numbers below, an asterisk will be used to indicate them.

The presence of a phenyl substituent at the C9* atom in molecules 3b and 3k (Fig. 6) suggests conjugation between the π-systems of the chromone fragment and the side benzene ring. The X-ray analysis revealed that, in contrast to their geometry in solution or the gas phase, flavonols in a crystalline state are non-planar molecules. Thus, in molecules 3b and 3k, these fragments are rotated relative to each other (torsion angle C8*–C9*–C10*–C11* in Fig. 6) by 12.3(3)° in molecule 3b and by −17.3(4)° in molecule 3k, and the length of the C9*–C10* bond (1.468(2) Å) in molecule 3b and 1.463(3) Å in molecule 3k) is comparable to the average length of the C_sp^2^_–C_ar_ bond in conjugated systems 1.470 Å.^52^

The non-planarity of flavonol molecules in the solid state, *i.e.* the presence of some non-zero dihedral angles between the molecular fragments additionally diminishes the influence of substituents on the side ring regarding the electron density distribution in the chromone part of the molecule. Thus, the effect of substituents on the spectral properties of flavonols occurs only indirectly, mediated by conformational effects and the polarity of the surrounding environment. The latter is influenced by the parameters of the crystal lattice, which can in turn be affected by the volumetric characteristics and positions of the substituents.

**Fig. 7:**
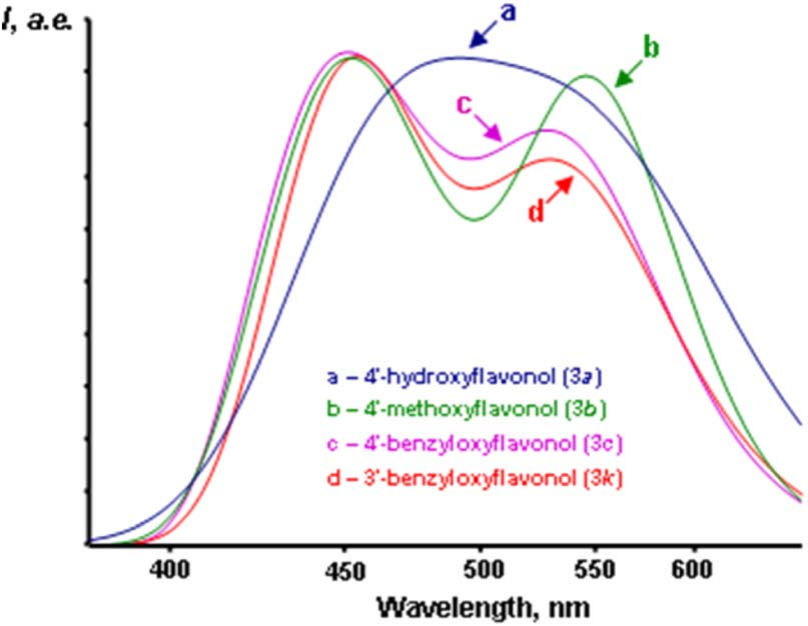
Fluorescence spectra of flavonols in the solid state.

Unlike the fluorescence spectra of flavonol solutions, which show only one emission band from the T* phototautomer, the spectra of flavonols in the solid state exhibit two emission bands (Fig. 7).

The short-wavelength fluorescence bands of the N* form in most flavonols typically range from 450 to 460 nm (approximately 22 200–21700 cm^−1^). However, in compounds 3d, 3e, and 3j, these bands are slightly shifted toward the long-wavelength region. It is important to note the large Stokes shifts of fluorescence, which range from 4800 to 6900 cm^−1^. This range is approximately 1500 to 2500 cm^−1^ greater than that observed in most fluorophores. This discrepancy likely results from significant energy costs associated with the structural relaxation of the excited molecule within a rigid crystal lattice.

The fluorescence bands of the T* form are centered between 540 and 560 nm (corresponding to 18 550–17850 cm^−1^). These bands are shifted by 20 to 30 nm toward the long-wavelength region of the spectrum compared to the fluorescence bands of the same form in the solutions. In the solid state, the Stokes shifts of the fluorescence of the flavonol phototautomer are smaller than those in the solutions, measuring between 8800 and 9730 cm^−1^. This suggests the absence of a significant structural relaxation of the phototautomer in the excited state.

No dependence was found between the positions of the N* and T* bands in the fluorescence spectra of crystalline flavonols and the nature or position of the substituents in the side ring.

The appearance of the short-wavelength N* emission band evidences that the intramolecular proton transfer is partially suppressed. The side ring substituents have weak electronic effects, and the non-planar geometry of the molecules suggests that the side rings have a minimal impact on proton transfer kinetics. Another factor contributing to the reduced efficiency of the excited-state intramolecular proton transfer is the weakening of the intramolecular hydrogen bond necessary for the proton transfer between the carbonyl group and the 3-hydroxy group of chromone.

**Fig. 8:**
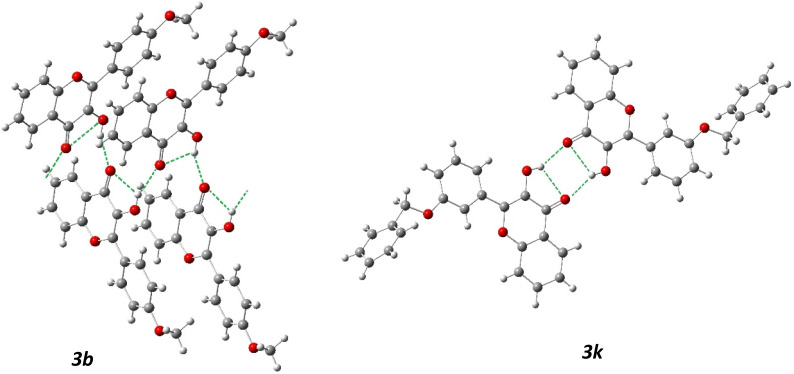
Crystalline structure of 4′-methoxy (3b) and 3′-benzyloxy (3k) flavonols. Intramolecular and intermolecular hydrogen bonds are shown with green dashed lines.

As illustrated in Fig. 8, the hydrogen atoms of the 3-hydroxy group form bifurcated hydrogen bonds. According to Etter's rules,^53,54^ the formation of intramolecular hydrogen bonds is preferred over intermolecular hydrogen bonds. However, the hydrogen bond that closes the five-membered ring is not sufficiently effective. This likely leads to the hydroxyl group in all three structures acting as a proton donor in two hydrogen bonds simultaneously: the intramolecular O_3_*–H⋯O_2_* and the intermolecular O_3_*–H⋯O_2_′*. The O_2_* atom, in turn, serves as a proton acceptor in both hydrogen bonds.

The nature of the primary structural motif formed by the intermolecular hydrogen bonds varies. In structure 3b, hydrogen-bonded chains are formed, while in structures 3c and 3k, centrosymmetric hydrogen-bonded dimers are established (see Fig. 8). It is important to note that the formation of bifurcated hydrogen bonds weakens each bond, as demonstrated by their characteristics (refer to Table 6).

**Table 6:** Geometric characteristics of hydrogen bonds in structures 3b and 3k

Hydrogen bond	Symmetry operation	Geometric characteristics		
		H⋯O, Å	O⋯O, Å	O–H⋯O, deg
**Structure 3b**				
O3*–H⋯O2*		2.23	2.677(2)	113.30
O3*–H⋯O2′*	1 − *x*,0.5 + *y*,0.5 − z	1.98	2.753(2)	153.6

**Structure 3k**				
O3*–H⋯O2*		2.27	2.712(3)	113.5
O3*–H⋯O2’*	−x, −y, 1 − z	2.01	2.751(3)	146.0

Since the formation of multi-center hydrogen bonds weakens the intramolecular hydrogen-bond component, it leads to a decrease in the rate of phototautomerization. Consequently, the rate of the proton transfer becomes comparable to the rate of emission of the original form N*, which results in the appearance of the corresponding short-wavelength band N* in the spectra.

The fluorescence intensity ratio of the initial form (N*) to the phototautomer (T*), represented as *I*_N_/*I*_T_, indicates that for most compounds, the emission intensity of form N* is greater than that of form T*. In several flavonols, the intensity *I*_N_ can exceed IT by 10–20%. However, most compounds show low ESIPT efficiency with the luminescence intensity of form N* surpassing that of form T* by 1.5 to 2.5 times. An analysis of the *I*_N_/*I*_T_ values reveals that the ratio of forms N* and T* in the excited state does not depend on the position and nature of the substituents. Nonetheless, a significant slowdown in ESIPT is primarily observed in flavonols with bulky benzyl substituents. This suggests that the spectral properties of these compounds must be determined by the characteristics of their crystal.

### Binding interactions of flavonols with β-glucosidase

Fluorescent flavonols serve as promising molecular probes for investigating protein-ligand interactions, with the goal of understanding the mechanisms of enzyme action.^55,56^ They also provide a theoretical foundation for the design and discovery of new regulators of enzymatic activity.^55,57,58^ Recently, we demonstrated that the ESIPT fluorescence of flavonols is highly sensitive to binding interactions with proteins, making it an effective tool for probing protein structure and their hydrophobic pockets.^59,60^ Furthermore, incorporating bulky alkyl and benzoyl groups into the 2-phenyl ring of flavonols increases their hydrophobicity and enhances the binding affinity to β-glucosidase, allowing for the inhibition of the enzyme's activity.^26^

**Fig. 9:**
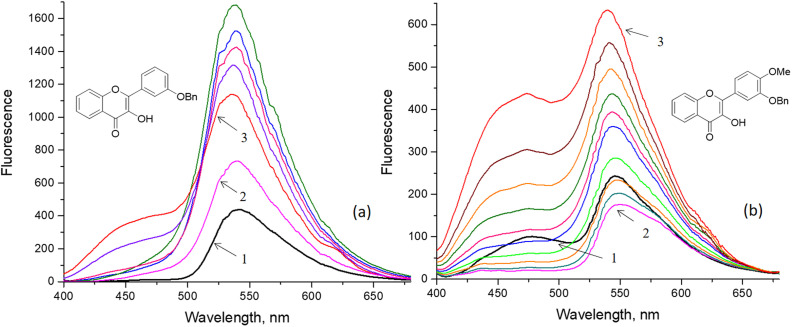
Fluorescence titration of flavonols 3k (a) and 3l (b) with β-glucosidase. The flavonol fluorescence was measured in the phosphate buffer pH 6.86 at 298 K after excitation at 380 nm. The arrows indicate the flavonol spectra: (1) in the absence of β-glucosidase, (2) after adding the first β-glucosidase aliquot, (3) in the presence of maximal concentration of β-glucosidase. The flavonol concentration of 2.3·10^−5^ M (a, b) was constant, whereas the β-glucosidase concentration was varied in a range from 1.1·10^−5^ up to 9.5·10^−4^ M (a) and from 2.2·10^−5^ up to 8.8·10^−4^ M (b).

In this study, we aimed to investigate the influence of C3′- and C4′-substitutions in flavonols on their protein binding affinity and selectivity. We examined the fluorescence properties of flavonols when interacting with β-glucosidase. Fig. 9 displays the results of fluorescence titration of flavonols 3k and 3l by varying concentrations of β-glucosidase in a phosphate buffer at pH 6.86. Despite their common use in biomedical applications, flavonols have low solubility in water.^61^ To address this issue, we dissolved them in the buffer by adding small aliquots of their DMSO stock solution. To explore the interaction between flavonols and the enzyme, we followed a fluorescence titration protocol similar to the one used in our recent study.^26^ In summary, during the titration, we maintained the concentration of flavonols at approximately 1–2 × 10^−5^ M, while gradually varying the concentration of glucosidase from 0 to 9.5 × 10^−4^ M.

As shown in Fig. 9, the fluorescence intensity of both flavonols 3k and 3l increases with higher concentrations of β-glucosidase. This phenomenon, which has also been observed with other flavonols, is attributed to a protein-induced “turn-on” effect.^26,39,58,59,62^ The increase in fluorescence can be explained by the reduction of water-induced fluorescence quenching, which occurs as the probes bind to and penetrate deeper into the water-free hydrophobic regions of biomacromolecules.^6,63^ In addition to the significant “turn-on” effect observed, there is also a noticeable redistribution of the normal-to-phototautomer emission intensity. Specifically, the contribution of the normal form, represented by the short-wavelength band around 450 nm, gradually increases. This dual-band redistribution is more prominent in the case of flavonol 3l, indicating a possible alteration in its binding mode to the β-glucosidase protein (see Fig. 9b).

The strong fluorescence of the normal form suggests that the ESIPT process is somewhat inhibited in the protein-bound state of flavonols 3k and 3l. Several factors could explain this, including: (i) the formation of intermolecular hydrogen bonds with protein residues, or (ii) the disruption of the intramolecular hydrogen bond between the 3-OH group and the 4O carbonyl oxygen due to steric constraints within the protein-binding pocket. Additionally, it is possible that both mechanisms contribute to the ESIPT-sensitive behavior of the flavonols. This implies that even small modifications in the peripheral side ring of the chromone moiety could significantly influence ligand–protein interactions, including their strength and selectivity.

### ADMET properties

The physicochemical properties of molecular probes influence their water solubility, lipophilicity, and binding affinity to organized hydrophobic environments, such as micelles, lipid membranes, plasma proteins and other biomacromolecules. In the context of *in vitro* and in-cell studies, a proper balance of these properties is crucial for governing the adsorption, distribution, metabolism, and pharmacokinetics of flavonols used as ESIPT probes. Therefore, a thorough understanding of these properties, referred to as ADMET (Absorption, Distribution, Metabolism, Excretion, and Toxicity), along with their measurement and prediction, is essential for successful probe design and development.^64^

Probe's lipophilicity affects its permeability across cell membranes, the partitioning and distribution of a drug-like molecule, as well as its metabolism and renal excretion.^65^ Our fluorescence titration experiments shown in Fig. 9 demonstrated that flavonol binding to the protein was primarily driven by the hydrophobic effect because of their high liphophilicity. Therefore, we first tried to see if there is some correlations between spectral changes seen in Fig. 9 and liphophilicity of the studied flavonols.

Lipophilicity is defined as the logarithm of the octanol–water partition coefficient (log *P*_o/w_) that reflects the ability of a molecule to dissolve in the liphophilic environment, such as a lipid membrane.

Table 7 summarizes calculated consensus log *P* for model flavonol 3 and studied flavonols 3a–m. Table 7 illustrates that the gradual introduction of methoxy and benzoyl groups into the 2-phenyl ring increases the log *P*_o/w_ value from 2.84 for the unsubstituted flavonol to as high as 5.87 for the compound 3m. It is also noteworthy that empirical *in silico* predictions of log *P*_o/w_ parameters are not sensitive to the specific positions of substitutions. For instance, both 3j and 3l were predicted to have the same log *P*_o/w_ value of 4.33. To thoroughly investigate the molecular-level details of how variations in the C3′ and C4′-substituents fine-tune protein-ligand binding interactions, computational tools, such as molecular docking, that account for the implicit structure of proteins should be utilized.

**Table 7:** Calculated ADMET properties of the studied flavonols^a^

Flavonol	3′-R_1_	4′-R_2_	Mw (g mol^−1^)	TPSA (Å2)	Number of H-bond donors	Number of H-bond acceptors	Consensus log *P*_o/w_*
3	H	H	238.2	50.4	1	3	2.84
3a	H	OH	254.2	70.7	2	4	2.77
3b	H	OCH_3_	268.3	59.7	1	4	3.02
3c	H	OBn	330.3	59.7	1	4	4.57
3d	OH	H	254.2	70.7	2	4	2.77
3e	OH	OH	270.2	90.9	3	5	2.34
3f	OH	OCH_3_	284.3	79.9	2	5	2.62
3g	OCH_3_	H	268.3	59.7	1	4	3.02
3h	OCH_3_	OH	284.3	79.9	2	5	2.62
3i	OCH_3_	OCH_3_	298.3	68.9	1	5	2.85
3j	OCH_3_	OBn	360.4	68.9	1	5	4.33
3k	OBn	H	330.3	59.7	1	4	4.57
3l	OBn	OCH_3_	360.4	68.9	1	5	4.33
3m	OBn	OBn	422.4	68.9	1	5	5.87

a Consensus log *P*_o/w_ was estimated based on set of *in silico* predicting tools, such as PreADME, Molinspiration, XLOGP3, ALOGPS 2.1, ChemDoodle, pkCSM, and Osiris Property Explorer, respectively.

### Molecular docking calculations

Due to the unavailability of the high-resolution 3D structure of the β-glucosidase from commercial almonds used in our experimental studies,^66^ we turned to X-ray structures of β-glucosidases from other sources. The β-glucosidase family includes a variety of enzymes that exhibit different activities and are widely distributed across many living organisms.^67^ These enzymes share the common ability to hydrolyze β-glucosidic linkages found in disaccharides, oligosaccharides, and conjugated saccharides.

**Fig. 10:**
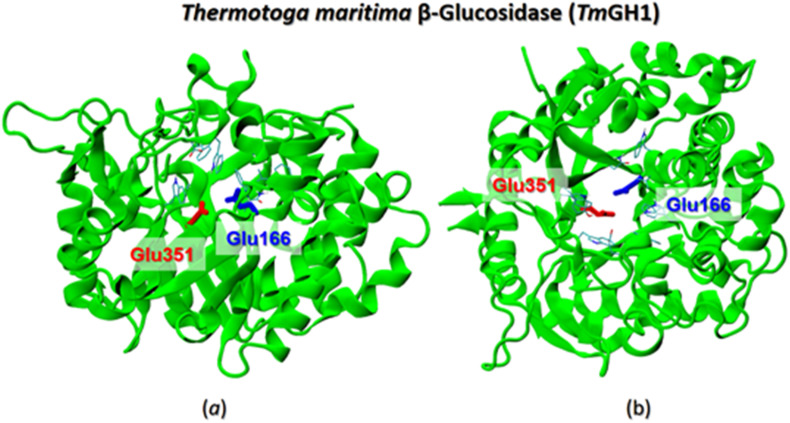
The X-ray structure of *Thermotoga maritima* β-glucosidase (PDB 1OD0)^47^ shown in a side (a) and top (b) views, respectively. The catalytic Glu166 and Glu351 residues for are shown by color-coded sticks.

Fig. 10 illustrates the X-ray structure of the β-glucosidase from *Thermotoga maritima* (TmGH1). Although there are some variations in the primary structure at the peripheral regions of the protein, all enzymes within the glucosidase family exhibit a high sequence identity of over 90%. The main common features of these enzymes include: (i) the presence of a deep hydrophobic pocket that can accommodate substrate molecules during cellulolytic hydrolysis (Fig. 10a), and (ii) a catalytic active site composed of two glutamate (Glu) residues situated in close proximity to each other (see Fig. 10a and b).

For our molecular docking analysis of flavonol–glucosidase interactions, we selected β-glucosidases from four different sources: *Paenibacillus polymyxa* β-glucosidase B (BglB),^45^*Raucaffricine* β-glucosidase (rBG),^46^*Thermotoga maritima* β-glucosidase (TmGH1),^47^ and human cytosolic β-glucosidase (hCBG).^48^ The X-ray 3D structures of these proteins have been well resolved, so that some of them already used as receptor models for molecular docking calculations.^26,39,62,68–70^

The structural and energetic characteristics of the interactions between flavonols 3k and 3l, and β-glucosidases were analyzed using molecular docking calculations, which are summarized in Table 8.

**Table 8:** The binding affinity of flavonols with β-glucosidases from various sources estimated by molecular docking calculations

Flavonol	3′-R_1_	4′-R_2_	Binding affinity, kcal mol^−1^			
			Human cytosolic β-glucosidase (PDB 2JFE)	*Paenibacillus polymyxa* β-glucosidase (PDB 2O9R)	*Thermotoga maritima* β-glucosidase (PDB 1OD0)	*Raucaffricine* β-glucosidase (PDB 4A3Y)
3	H	H	−8.8	−8.3	−8.5	−8.7
3a	H	OH	−9.2	−8.5	−8.9	−8.5
3b	H	OCH_3_	−9.3	−8.9	−8.6	−8.8
3c	H	OBn	−10.4	−9.1	−9.3	−9.3
3d	OH	H	−9.0	−8.6	−8.9	−9.5
3e	OH	OH	−9.6	−9.0	−8.8	−9.0
3f	OH	OCH_3_	−8.6	−8.4	−8.2	−9.0
3g	OCH_3_	H	−8.9	−8.9	−8.7	−9.4
3h	OCH_3_	OH	−9.0	−8.7	−8.9	−9.3
3i	OCH_3_	OCH_3_	−8.8	−8.7	−8.6	−8.0
3j	OCH_3_	OBn	−10.4	−9.4	−9.3	−9.3
3k	OBn	H	−10.6	−10.0	−9.9	−9.8
3l	OBn	OCH_3_	−10.6	−9.7	−9.4	−9.2
3m	OBn	OBn	−11.9	−11.1	−11.0	−10.2

The results indicate that all examined flavonols exhibit strong binding affinities, with values exceeding −8.5 kcal mol^−1^. Furthermore, flavonols can penetrate deeply into the central cavity of β-glucosidase and bind closely to the catalytic glutamic acid (Glu) residues.

**Fig. 11:**
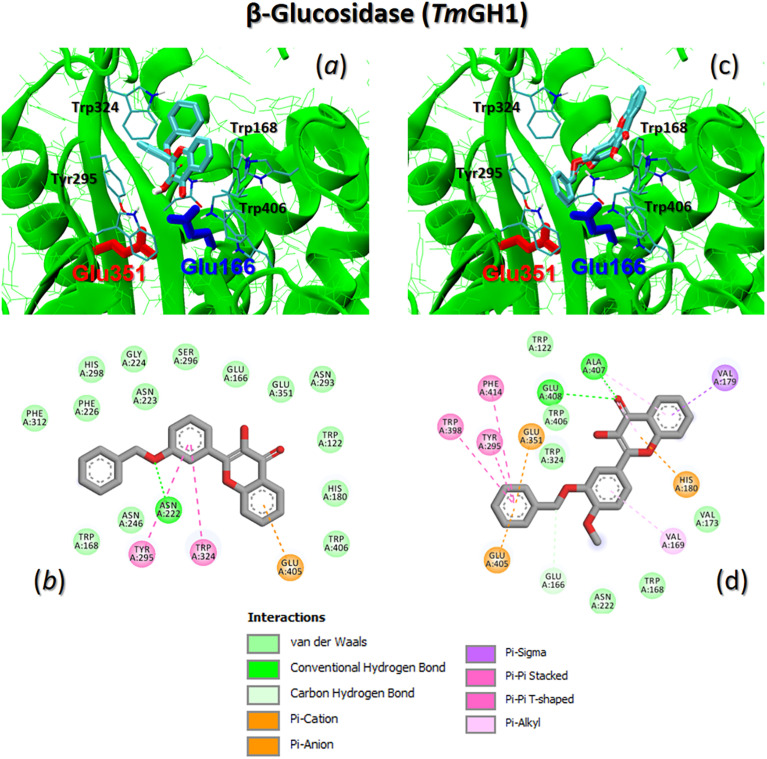
The best binding mode of flavonols 3k (a and b) and 3l (c and d) to β-glucosidase TmGH1 (PDB 1OD0). The catalytic residues Glu166 and Glu351 are shown as color-coded sticks. Some key enzyme interacting residues are also shown. (b and d) 2D interaction maps of selected flavonols with key protein residues.

Examples of binding modes for flavonols are illustrated in Fig. 11, specifically for β-glucosidase TmGH1. Molecular docking studies indicate that the binding affinity of compounds 3kand 3lto β-glucosidase depends on the specific characteristics of the peripheral substituents located at the C3′- and C4′-positions of the flavonol's phenyl ring.

In the case of flavonol 3k, it binds deeper into the enzyme pocket, with the 3OH-chromone moiety favoring a position close to the catalytic residue Glu166. The bulky 3′-benzyl ring protrudes outward from the enzyme pocket (see Fig. 11a). In contrast, the introduction of an additional 4′-methoxy group results in a reversal of the binding mode. For flavonol 3l, the 2-phenyl ring, featuring the bulky 3′-benzyl and 4′-methoxy groups, occupies the catalytic pocket, displacing the 3OH-chromone moiety outwards (see Fig. 11c).

Fig. 11b and d illustrate 2D interaction maps of flavonols with β-glucosidase TmGH1. Flavonol 3k binds to TmGH1 through non-covalent interactions, including π-π stacking of the 2-phenyl ring with TYR295 and TRP324. It also involves short-range π-anion interactions between the aromatic ring of the chromone and GLU405, as well as hydrogen bonding between the 3′-benzyl oxygen atom and ASN222 (Fig. 11b). In contrast, flavonol 3l exhibits much more complex interaction patterns. The major contributions to its binding include classical π-π stacking or π-π T-shaped stacking of the 3′-benzyl ring with TYR295, TRP298, and PHE414. Additionally, there are π-anion interactions of the 3′-benzyl ring with GLU405 and catalytic GLU351, along with hydrogen bonding of the 2O carbonyl oxygen atom with ALA407 and GLU408 (Fig. 11d).

### Summary and perspectives

Natural flavonols, including compounds like quercetin, morin, fisetin, and galangin, are among the most abundant classes of metabolic polyphenols. They exhibit a wide range of pharmacological activities beneficial to human health.^1,2^ Many flavonols also display unique dual ESIPT-emission fluorescence, which makes them sensitive to changes in their microenvironment.^6,7^ This property offers exciting opportunities for sensing and detecting metal ions, anions, small ligands, and biomacromolecules.^3,24,58,71^ However, the diverse structures of flavonols—characterized by variations in the number and position of hydroxyl groups, as well as various chemical modifications—complicate the relationship between their structure and fluorescence.^23,72^ As a result, using them as fluorescent probes can often be challenging.^58^

This study aims to clarify the influence of hydroxy, methoxy, and benzyl groups at the C3′ and C4′ positions of the 2-phenyl side ring of flavonols. We focus on examining their ESIPT fluorescence, crystal packing, physicochemical properties, and ADMET characteristics. A series of flavonols were synthesized, and their structures were characterized using NMR and MS analysis. We systematically investigated the fluorescence properties of the synthesized flavonols in aprotic solvents, analyzing the relationships between their structures and properties. Our main findings suggest that the nature and position of substituent groups in flavonols significantly influence their crystal packing in the solid state. We discovered that the molecular arrangement in the crystal lattice could be affected by intra- and intermolecular hydrogen bonding ratio, which in turn affects the ESIPT dual-band ratio. Furthermore, by employing fluorescence titration and molecular docking calculations, we explored how the introduction of a bulky benzyl moiety and the alteration of its position between C3′- and C4′- can impact the binding interactions of flavonols with β-glucosidases. We believe our findings shed light on the structure–fluorescence relationship in flavonols and open up new possibilities for the design of innovative flavonol-based probes.

## Author contributions

Oleksii O. Demidov: investigation, writing – original draft, writing – review and editing. Liudmyla V. Chepeleva, Svitlana V. Shishkina: investigation, writing – original draft, writing – review and editing. Eugene S. Gladkov: investigation, writing – original draft. Alexander V. Kyrychenko: investigation, conceptualization, writing – original draft, writing – review and editing. Rostyslav P. Linnik: conceptualization, writing – original draft. Alexander D. Roshal: supervision, conceptualization, writing – original draft, writing – review and editing.

## Conflicts of interest

There are no conflicts to declare.

## Supplementary Material

RA-015-D5RA05790F-s001

RA-015-D5RA05790F-s002

## Data Availability

The additional data for this study are available from the corresponding author upon request. CCDC 2416411 (3b), 2416412 (3c) and 2416347 (3k) contain the supplementary crystallographic data for this paper.^73*a*–*c*^ Supplementary information: the crystallographic data in Table S1, ^1^H and ^13^C NMR spectra of 3a–m in Fig. S1–S13 and mass spectra of 3a–m in Fig. S14–26. See DOI: https://doi.org/10.1039/d5ra05790f.
