# Supplementary material for: Influence of C3′- and C4′-substitutions on fluorescence, crystal packing, and physicochemical properties of flavonol
Source: RSC Adv. 2025 Sep 30;15(43):36300–18. doi: 10.1039/d5ra05790f (PMC12482225; doi:10.1039/d5ra05790f)
Supplement: RA-015-D5RA05790F-s001 [file RA-015-D5RA05790F-s001.pdf]

Supplementary Information (SI) for RSC Advances.
This journal is © The Royal Society of Chemistry 2025

Supporting Information

Influence of C3'- and C4'-Substitutions on Fluorescence, Crystal Packing, and Physicochemical Properties of Flavonol

Oleksii O. Demidov,[a] Liudmyla V. Chepeleva,[a] Svitlana V. Shishkina,[b,c]
Eugene S. Gladkov,[a,b] Alexander V. Kyrychenko[a,b],
Rostyslav P. Linnik[d] and Alexander D. Roshal[a]

[a.] Institute of Chemistry, V.N. Karazin Kharkiv National University, 4 Svobody Sq., Kharkiv 61022, Ukraine

[b.] State Scientific Institution "Institute for Single Crystals", National Academy of Sciences of Ukraine, 60 Nauky Ave., Kharkiv 61072, Ukraine

[c.] Institute of Organic Chemistry, NAS of Ukraine, 5 Akademik Kukhar Str., Kyiv 02066, Ukraine

[d.] Taras Shevchenko National University of Kyiv, Analytical Chemistry Department, 64/13 Volodymyrska Str., Kyiv 01601, Ukraine

CONTENTS:

Supplemental Table S1. The crystallographic data and experimental parameters for compounds **3b** and **3k**…………………..……........……......................... **2**

Figures S1-S13. ^{1}H and ^{13}C NMR spectra of flavonol **3a-m**………………………………. **3**

Figures S14-S26. Mass spectra of flavonol **3-m**……….……………………………………..**16**

Table S1. The crystallographic data and experimental parameters for compounds **3b** and **3k**.

Parameter	***3b***	***3k***
Unit cell		
a, Å	11.2635(6)	19.780(2)
b, Å	4.9880(3)	4.9187(6)
c, Å	22.0194(12)	19.121(2)
α, deg	90.0	90.0
β, deg	95.119(3)	115.035(7)
γ, deg	90.0	90.0
V, $Å^3$	1232.17(12)	1685.6(3)
F(000)	560	720
Crystal system	Monoclinic	Monoclinic
Space group	$P2_1/c$	$P2_1/c$
Z	4	4
T, K	173(2)	173(2)
μ, mm^{-1}	0.104	0.093
D_{calc}, g/cm^3	1.446	1.357
$2\Theta_{max}$, grad	50	50
Measured reflections	16340	21576
Independent reflections	2158	2973
R_{int}	0.0525	0.0781
Reflections with $F>4\sigma(F)$	1751	1920
Parameters	184	272
R_1	0.0417	0.0593
wR_2	0.1142	0.1688
S	1.042	1.047
CCDC number	2416411	2416347

^{1}H and ^{13}C NMR spectra of flavonol 3a-m

^{1}H NMR (500 MHz, DMSO-d_6)

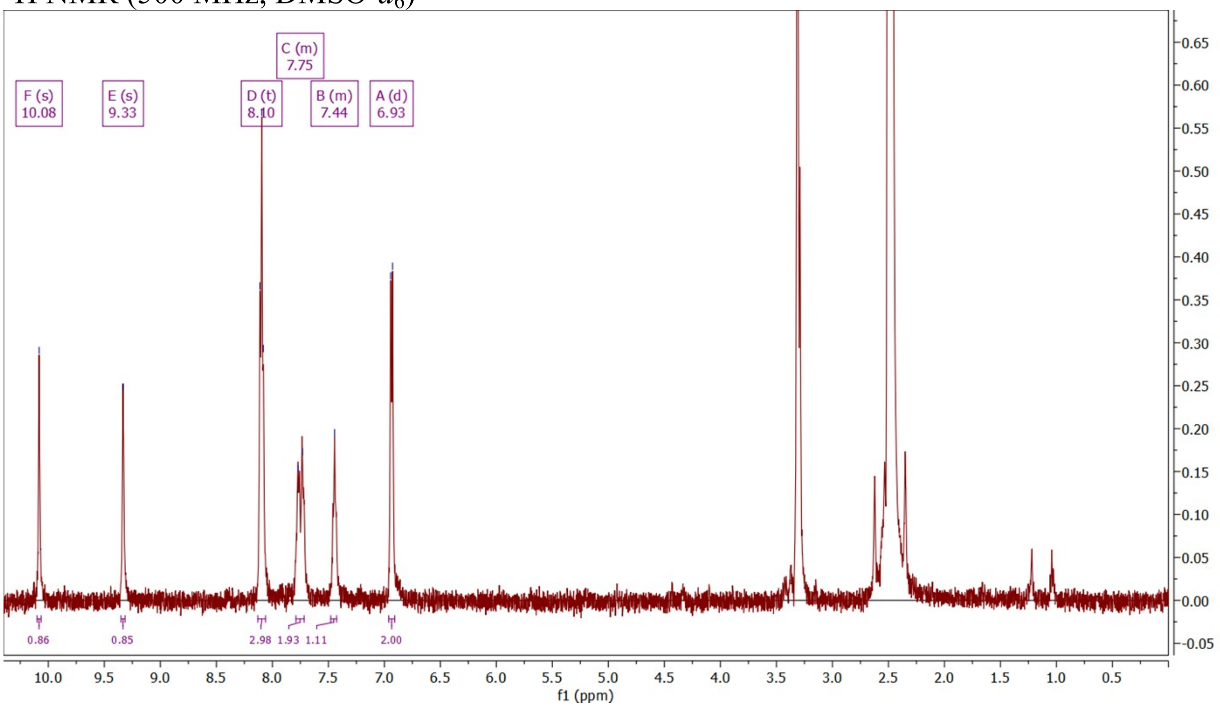

^{13}C NMR (126 MHz, DMSO-d_6)

^{13}C NMR (126 MHz, DMSO) δ 172.5, 159.1, 154.4, 146.1, 137.8, 133.3, 129.5, 124.7, 124.4, 122.0, 121.3, 118.2, 115.4.

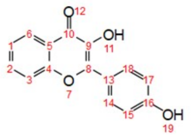

3-hydroxy-2-(4-hydroxyphenyl)-4H-chromen-4-one

Chemical Formula: $C_{15}H_{10}O_4$

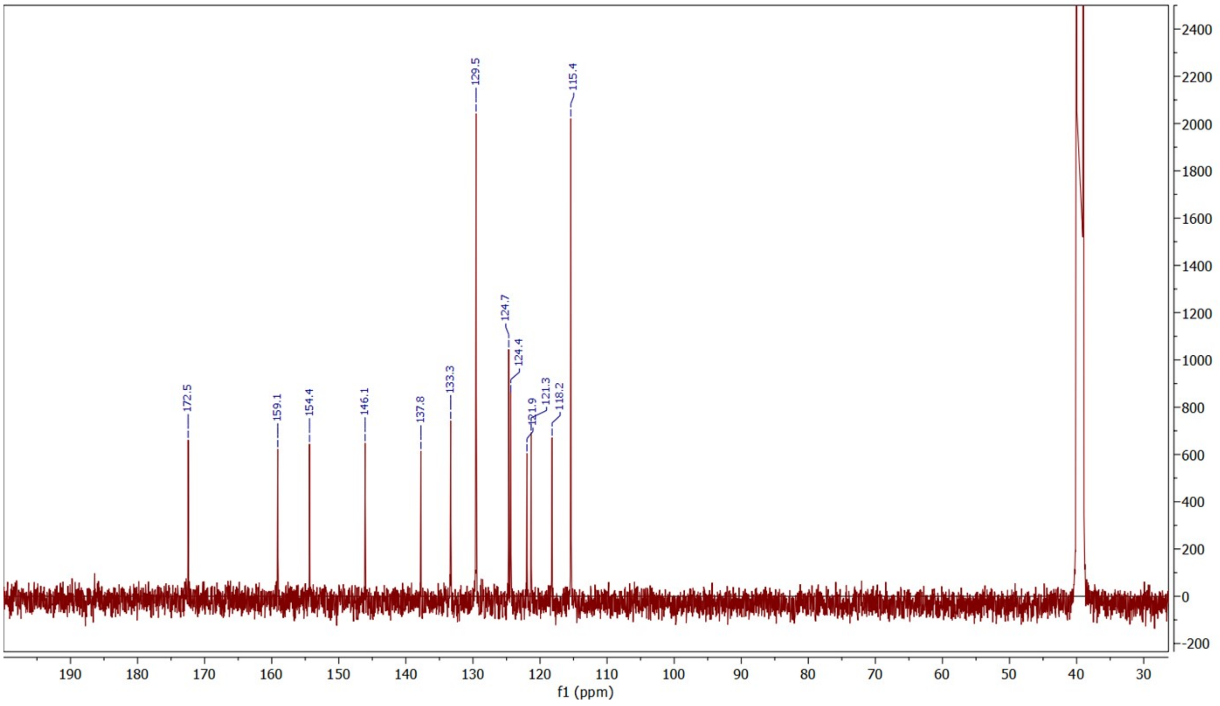

Figure S1. ^{1}H NMR (*top*) and ^{13}C NMR (*bottom*) spectra for flavonol **3a**

^{1}H NMR (500 MHz, DMSO-d_6)

^{1}H NMR (500 MHz, DMSO) δ 9.46 (d, J = 1.6 Hz, 1H), 8.20 (d, J = 8.6 Hz, 2H), 8.10 (d, J = 8.0 Hz, 1H), 7.82 – 7.72 (m, 2H), 7.45 (t, J = 7.4 Hz, 1H), 7.13 (d, J = 8.6 Hz, 2H), 3.84 (s, 3H).

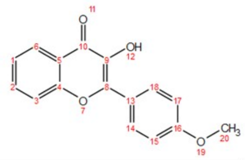

3-hydroxy-2-(4-methoxyphenyl)-4H-chromen-4-one

Chemical Formula: $C_{16}H_{12}O_4$

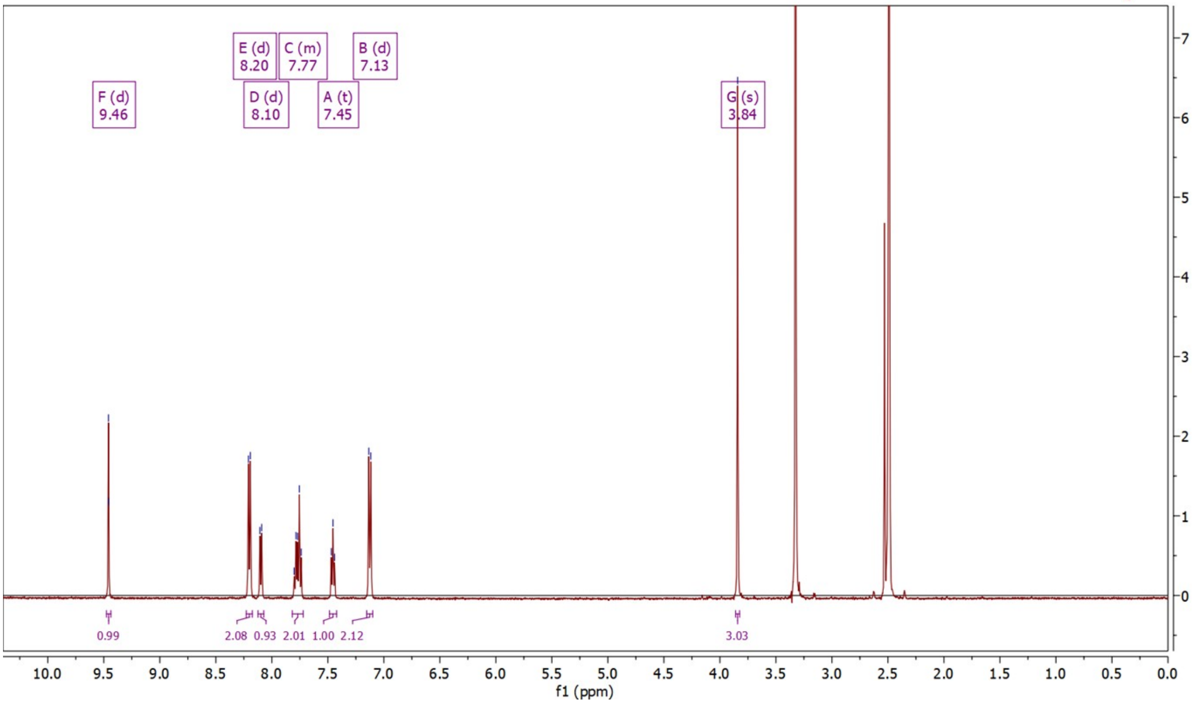

^{13}C NMR (126 MHz, DMSO-d_6)

^{13}C NMR (126 MHz, DMSO) δ 172.6, 160.4, 154.4, 145.5, 138.1, 133.4, 129.4, 124.7, 124.4, 123.5, 121.3, 118.3, 114.0, 55.3.

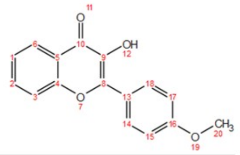

3-hydroxy-2-(4-methoxyphenyl)-4H-chromen-4-one

Chemical Formula: $C_{16}H_{12}O_4$

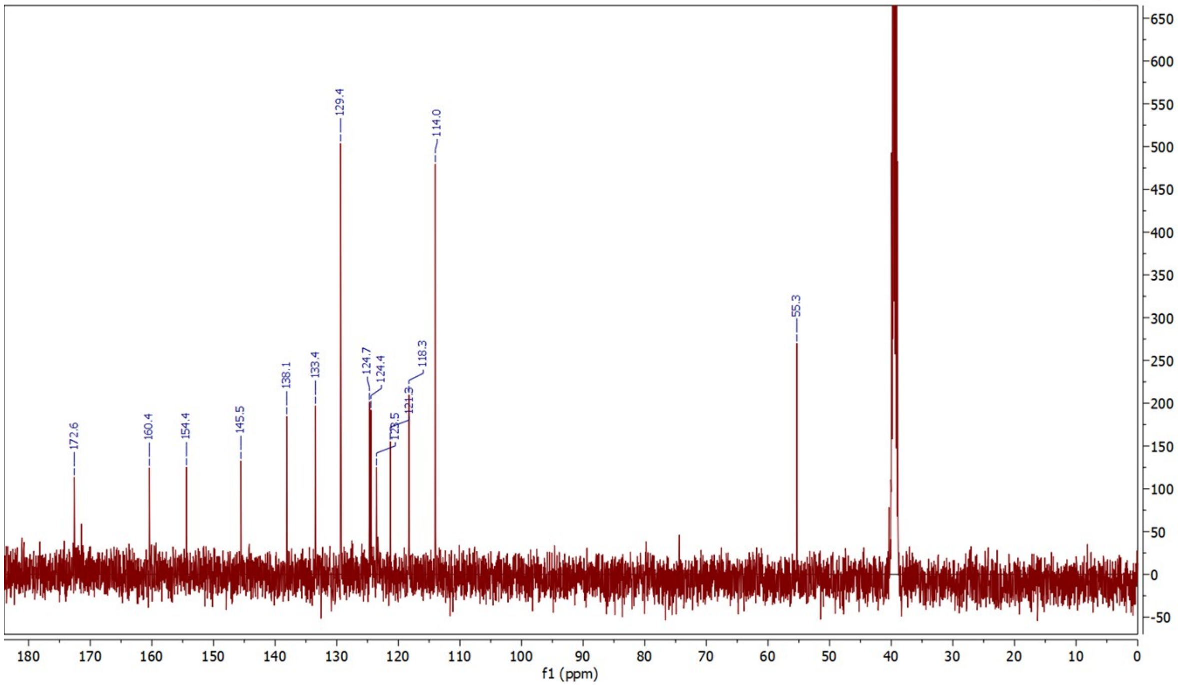

Figure S2. ^{1}H NMR (*top*) and ^{13}C NMR (*bottom*) spectra for flavonol **3b**

^{1}H NMR (500 MHz, DMSO-d_6)

^{1}H NMR (500 MHz, dmso) δ 9.31 (s, 1H), 8.22 – 8.15 (m, 2H), 8.09 (dt, J = 8.2, 1.8 Hz, 1H), 7.79 – 7.68 (m, 2H), 7.49 – 7.29 (m, 6H), 7.21 – 7.15 (m, 2H), 5.18 (s, 2H).

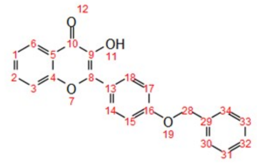

2-(4-(benzyloxy)phenyl)-3-hydroxy-4H-chromen-4-one
Chemical Formula: $C_{22}H_{16}O_4$

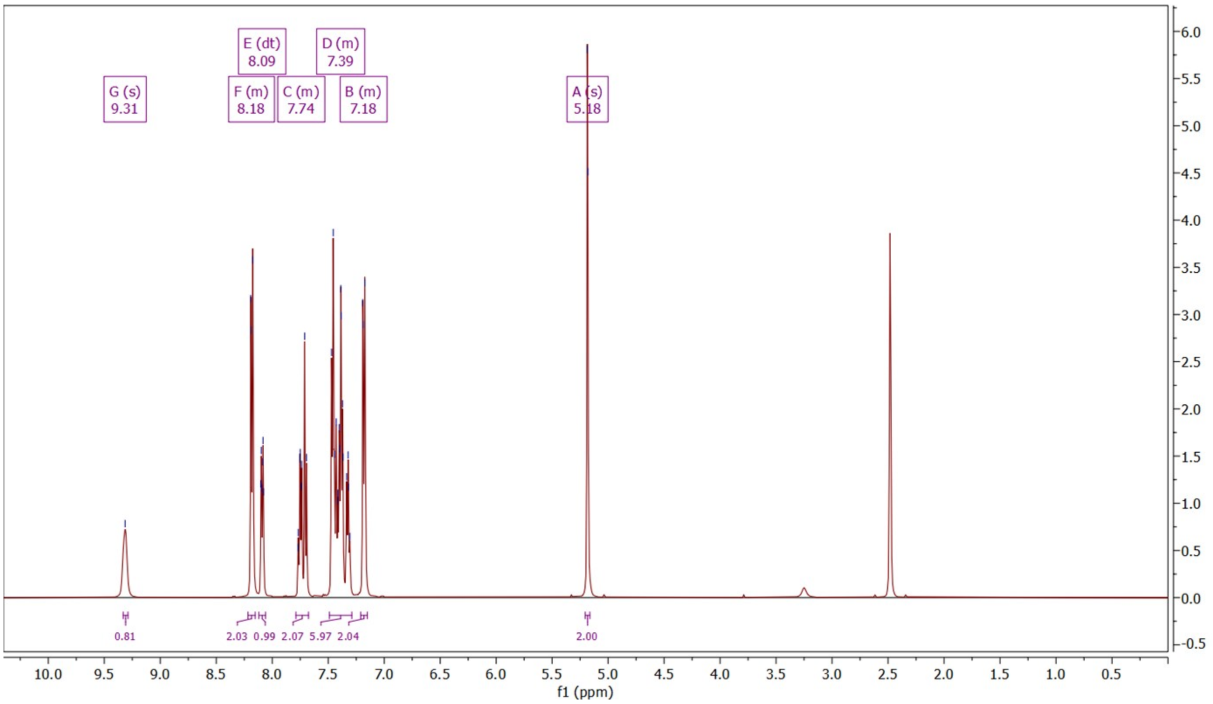

^{13}C NMR (126 MHz, DMSO-d_6)

^{13}C NMR (126 MHz, DMSO) δ 172.60, 159.48, 154.40, 145.46, 138.17, 136.64, 133.40, 129.34, 128.42, 127.90, 127.74, 124.68, 124.40, 123.76, 121.32, 118.25, 114.83, 69.36.

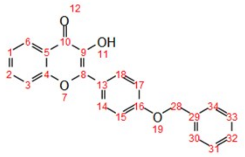

2-(4-(benzyloxy)phenyl)-3-hydroxy-4H-chromen-4-one
Chemical Formula: $C_{22}H_{16}O_4$

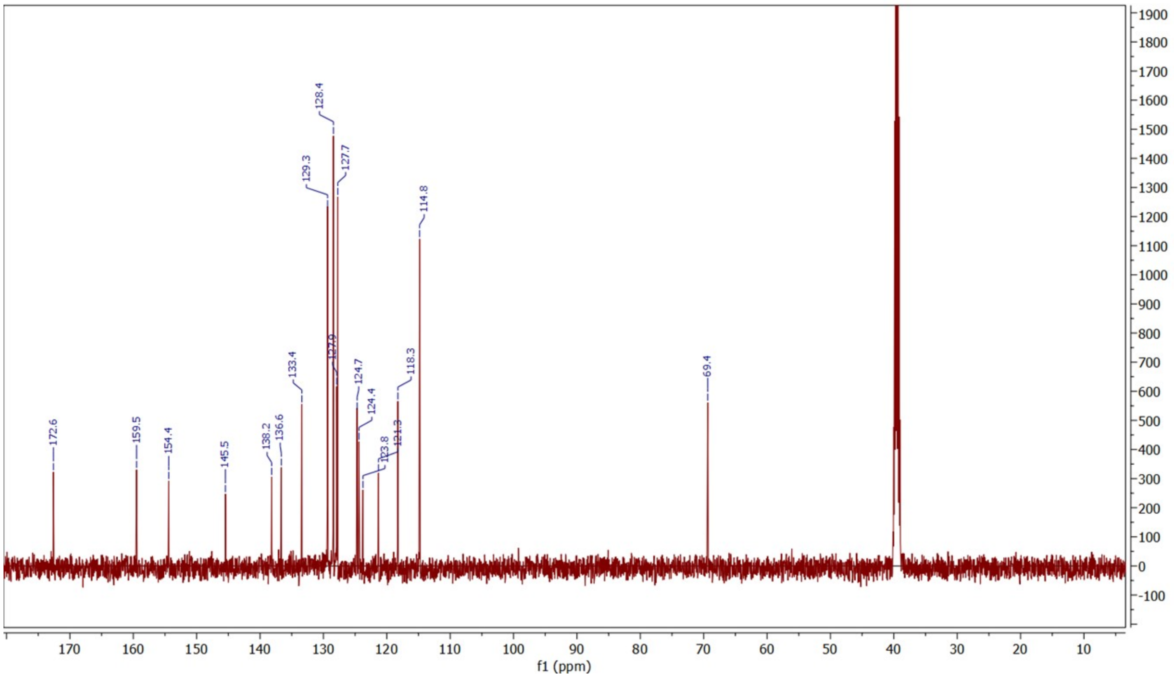

Figure S3.^{1}H NMR (*top*) and ^{13}C NMR (*bottom*) spectra for flavonol **3c**

^{1}H NMR (400 MHz, DMSO-d_6)

^{1}H NMR (400 MHz, DMSO) δ 9.71 (s, 1H), 9.56 (s, 1H), 8.11 (dd, J = 8.0, 1.7 Hz, 1H), 7.85 – 7.76 (m, 1H), 7.76 – 7.71 (m, 1H), 7.71 – 7.61 (m, 2H), 7.51 – 7.43 (m, 1H), 7.35 (t, J = 8.0 Hz, 1H), 6.93 – 6.86 (m, 1H).

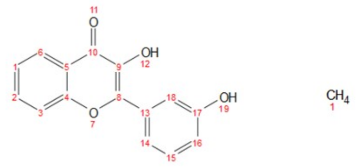

3-hydroxy-2-(3-hydroxyphenyl)-4H-chromen-4-one
Chemical Formula: $C_{15}H_{10}O_4$

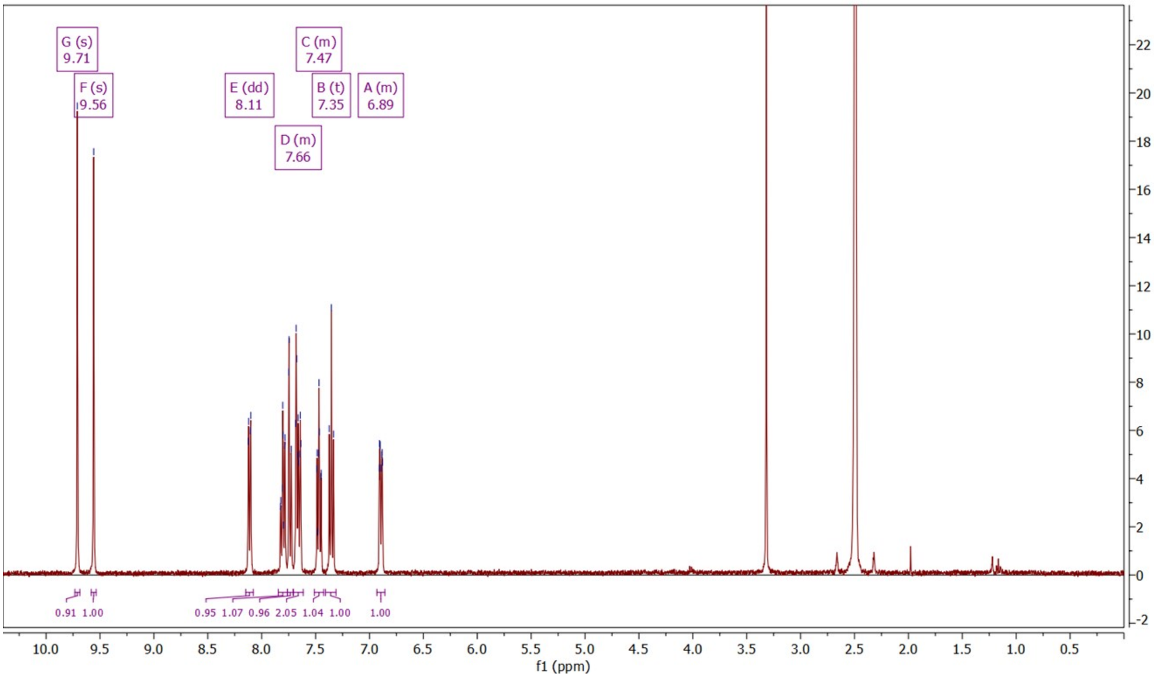

^{13}C NMR (101 MHz, DMSO-d_6)

^{13}C NMR (101 MHz, DMSO) δ 173.48, 159.59, 157.79, 155.02, 145.71, 139.56, 134.26, 132.90, 130.04, 125.30, 125.05, 121.74, 118.93, 117.54, 115.04.

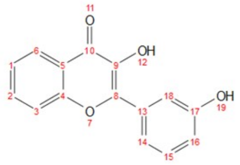

3-hydroxy-2-(3-hydroxyphenyl)-4H-chromen-4-one
Chemical Formula: $C_{15}H_{10}O_4$

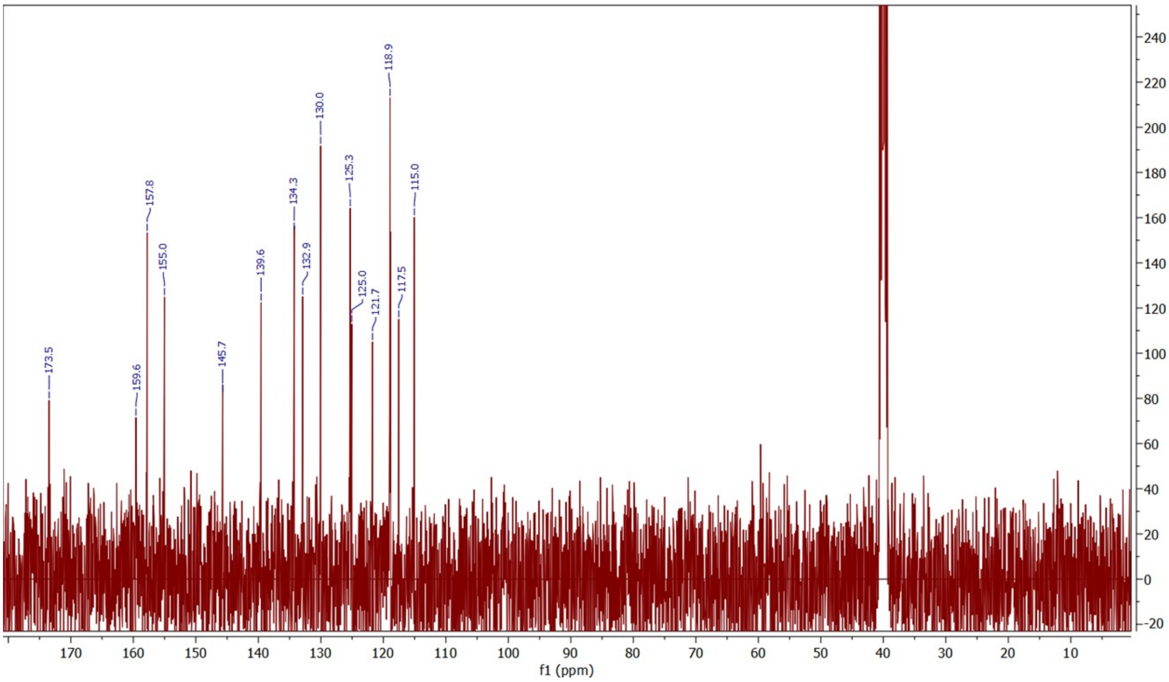

Figure S4. ^{1}H NMR (*top*) and ^{13}C NMR (*bottom*) spectra for flavonol **3d**

^{1}H NMR (400 MHz, DMSO-d_6)

^{1}H NMR (400 MHz, DMSO) δ 9.39 (s, 4H), 8.09 (dd, J = 7.9, 1.7 Hz, 1H), 7.81 – 7.72 (m, 2H), 7.69 (d, J = 8.4 Hz, 1H), 7.61 (dd, J = 8.4, 2.2 Hz, 1H), 7.43 (t, J = 7.4 Hz, 1H), 6.91 (d, J = 8.4 Hz, 1H).

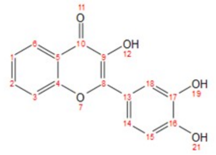

2-(3,4-dihydroxyphenyl)-3-hydroxy-4H-chromen-4-one
Chemical Formula: $C_{15}H_{10}O_5$

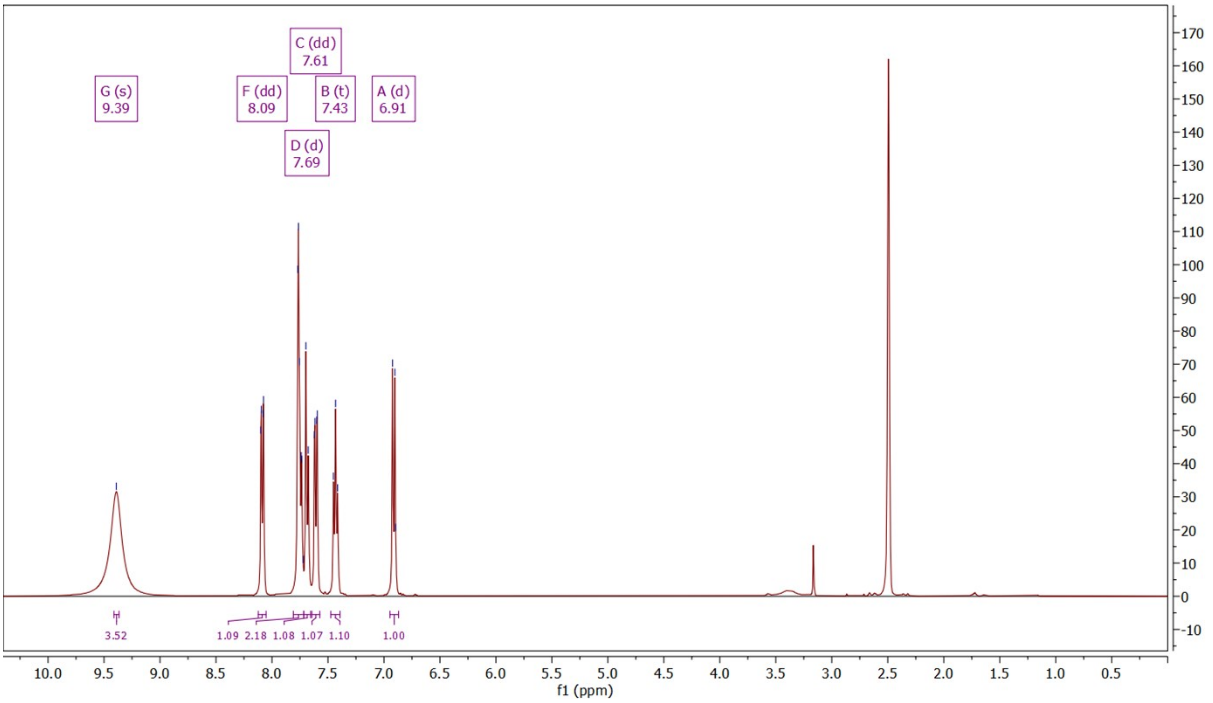

^{13}C NMR (101 MHz, DMSO-d_6)

^{13}C NMR (101 MHz, DMSO) δ 172.94, 154.82, 148.10, 146.58, 145.56, 138.36, 133.84, 125.19, 124.85, 122.76, 121.79, 120.47, 118.64, 116.09, 115.75.

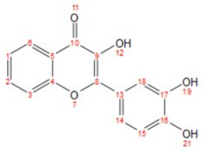

2-(3,4-dihydroxyphenyl)-3-hydroxy-4H-chromen-4-one
Chemical Formula: $C_{15}H_{10}O_5$

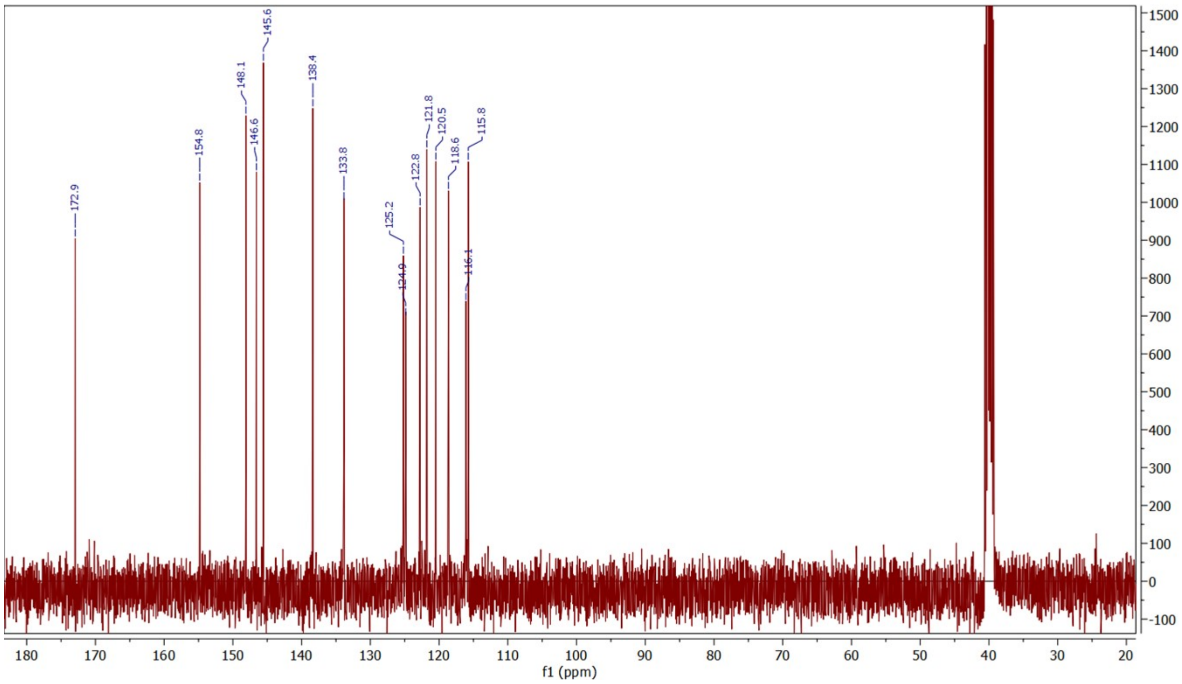

Figure S5. ^{1}H NMR (*top*) and ^{13}C NMR (*bottom*) spectra for flavonol **3e**

^{1}H NMR (400 MHz, DMSO-d_6)

^{1}H NMR (400 MHz, dmso) δ 9.25 (d, J = 14.1 Hz, 2H), 8.11 (dd, J = 8.0, 1.6 Hz, 1H), 7.82 – 7.67 (m, 4H), 7.45 (t, J = 7.4 Hz, 1H), 7.10 (d, J = 8.6 Hz, 1H), 3.86 (s, 3H).

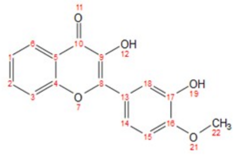

3-hydroxy-2-(3-hydroxy-4-methoxyphenyl)-4H-chromen-4-one
Chemical Formula: $C_{16}H_{12}O_5$

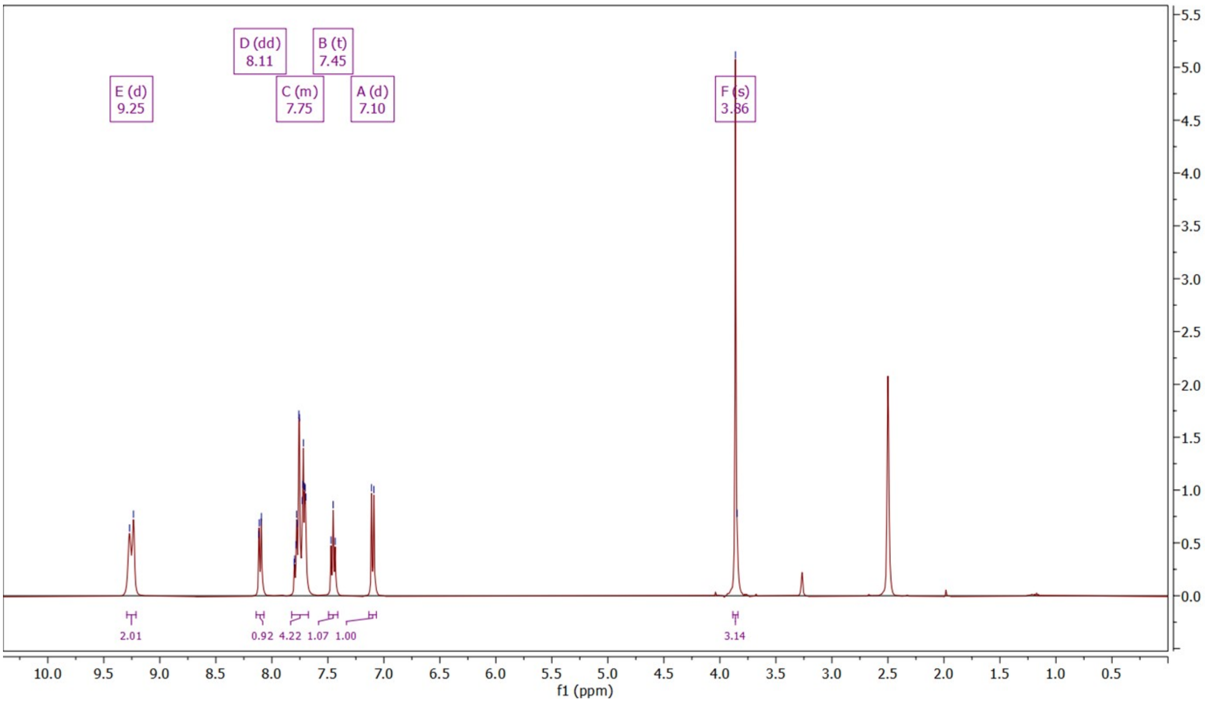

^{13}C NMR (126 MHz, DMSO-d_6)

^{13}C NMR (126 MHz, DMSO) δ 172.54, 154.37, 149.26, 146.20, 145.62, 138.17, 133.42, 124.69, 124.38, 123.74, 121.26, 119.73, 118.17, 114.80, 111.82, 55.61.

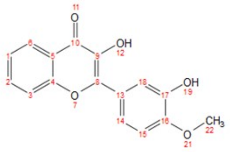

3-hydroxy-2-(3-hydroxy-4-methoxyphenyl)-4H-chromen-4-one
Chemical Formula: $C_{16}H_{12}O_5$

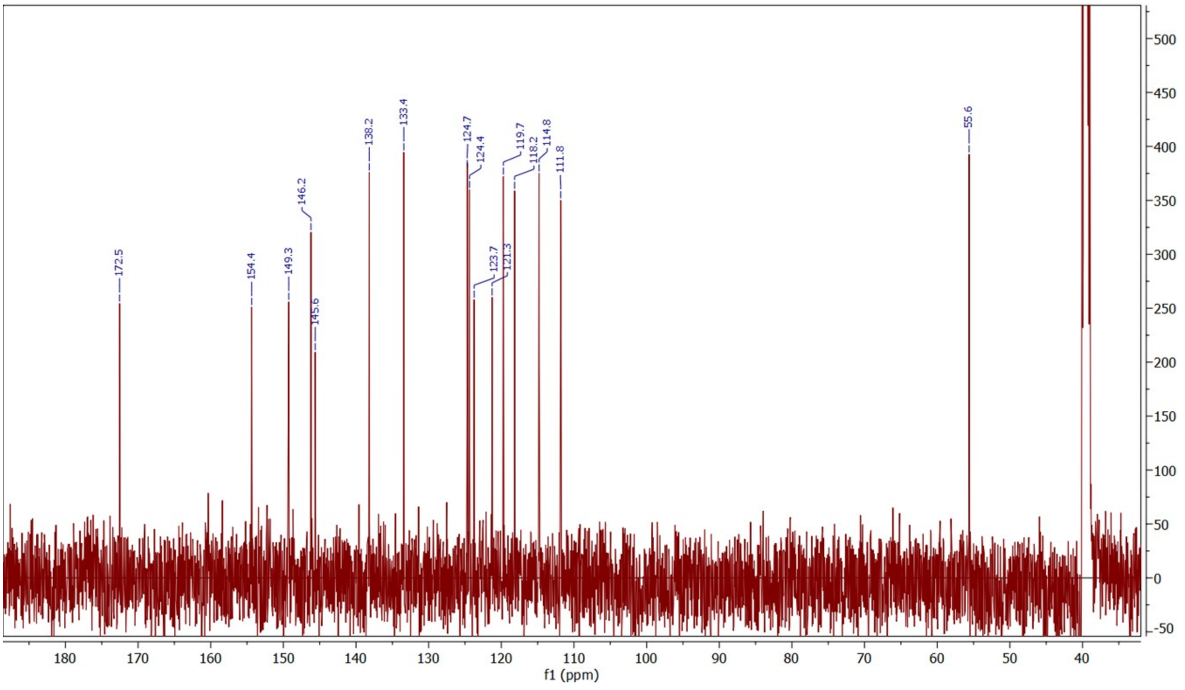

Figure S6.^{1}H NMR (*top*) and ^{13}C NMR (*bottom*) spectra for flavonol **3f**

^{1}H NMR (400 MHz, DMSO-d_6)

^{1}H NMR (400 MHz, DMSO) δ 9.66 (s, 1H), 8.15 – 8.08 (m, 1H), 7.90 – 7.69 (m, 4H), 7.54 – 7.37 (m, 2H), 7.10 (dd, *J* = 8.2, 2.6 Hz, 1H), 3.83 (s, 3H).

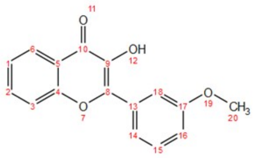

3-hydroxy-2-(3-methoxyphenyl)-4H-chromen-4-one
Chemical Formula: $C_{16}H_{12}O_4$

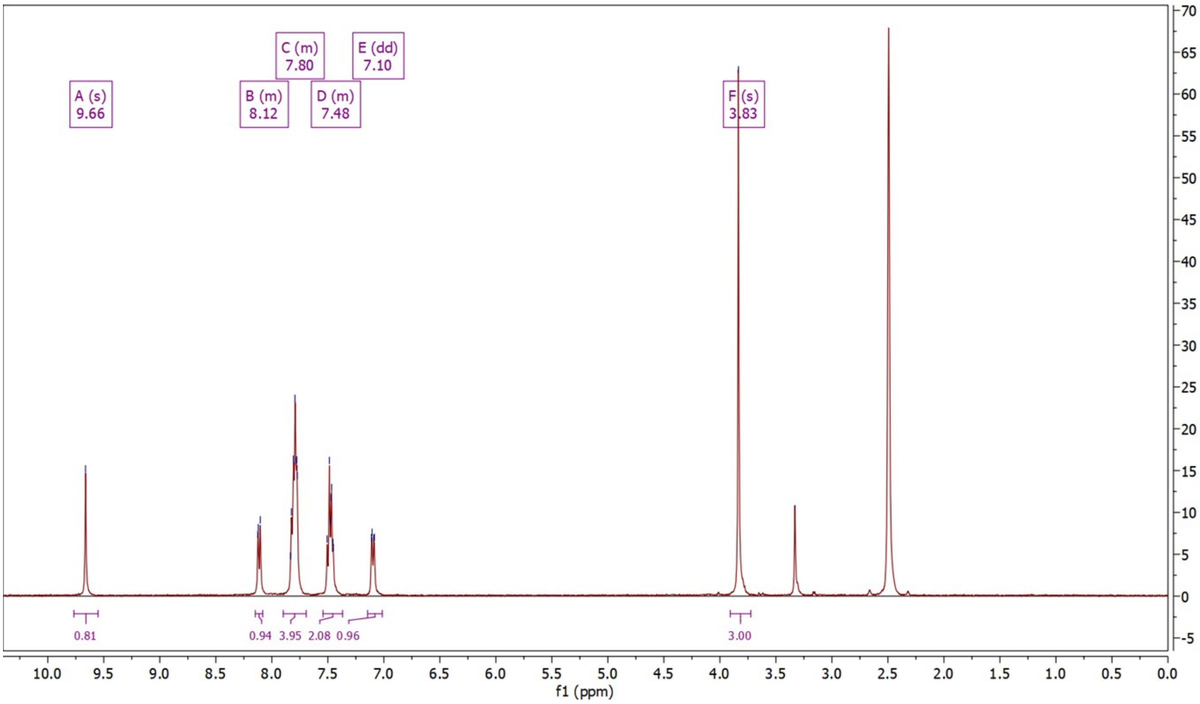

^{13}C NMR (101 MHz, DMSO-d_6)

^{13}C NMR (101 MHz, DMSO) δ 173.5, 159.7, 155.0, 145.3, 139.7, 134.3, 133.0, 130.2, 125.3, 125.1, 121.7, 120.5, 119.0, 115.7, 113.9, 55.7.

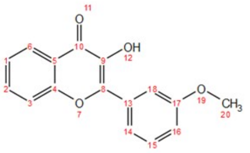

3-hydroxy-2-(3-methoxyphenyl)-4H-chromen-4-one
Chemical Formula: $C_{16}H_{12}O_4$

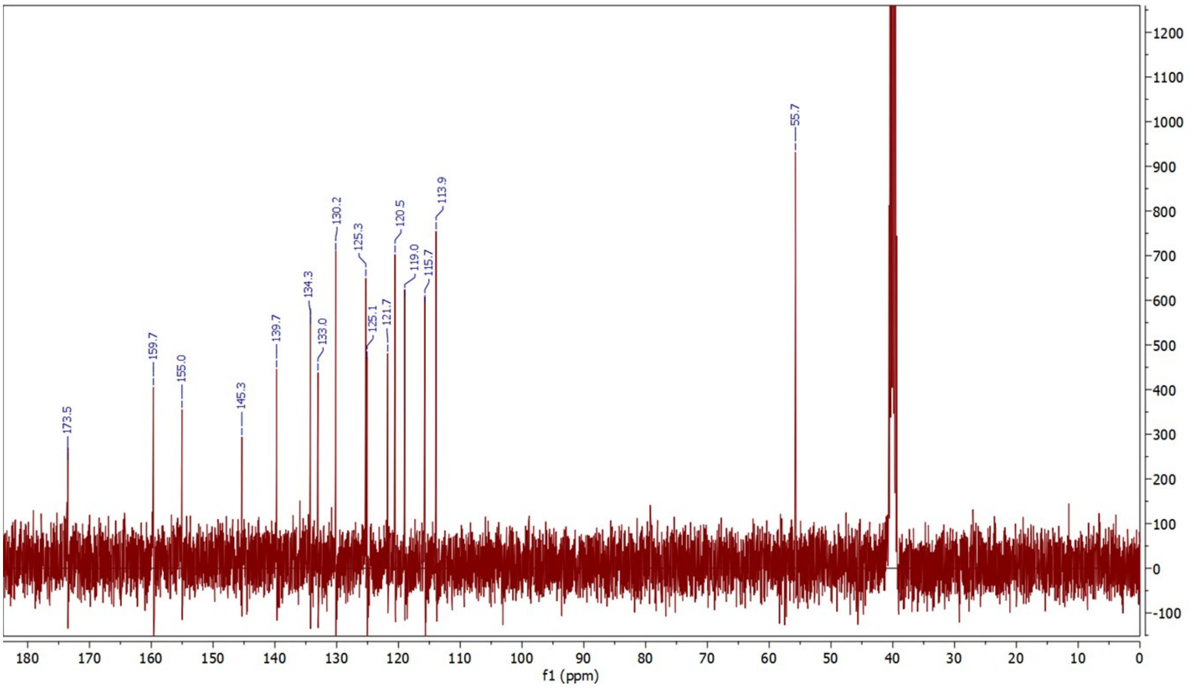

Figure S7. ^{1}H NMR (*top*) and ^{13}C NMR (*bottom*) spectra for flavonol **3g**

^{1}H NMR (400 MHz, DMSO-d_6)

^{1}H NMR (400 MHz,) δ 9.55 (s, 2H), 8.09 (d, J = 7.9 Hz, 1H), 7.91 – 7.66 (m, 4H), 7.50 – 7.40 (m, 1H), 6.96 (d, J = 8.5, 3.7 Hz, 1H), 3.86 (s, 3H).

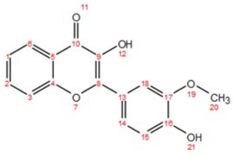

3-hydroxy-2-(4-hydroxy-3-methoxyphenyl)-4H-chromen-4-one
Chemical Formula: $C_{16}H_{12}O_5$

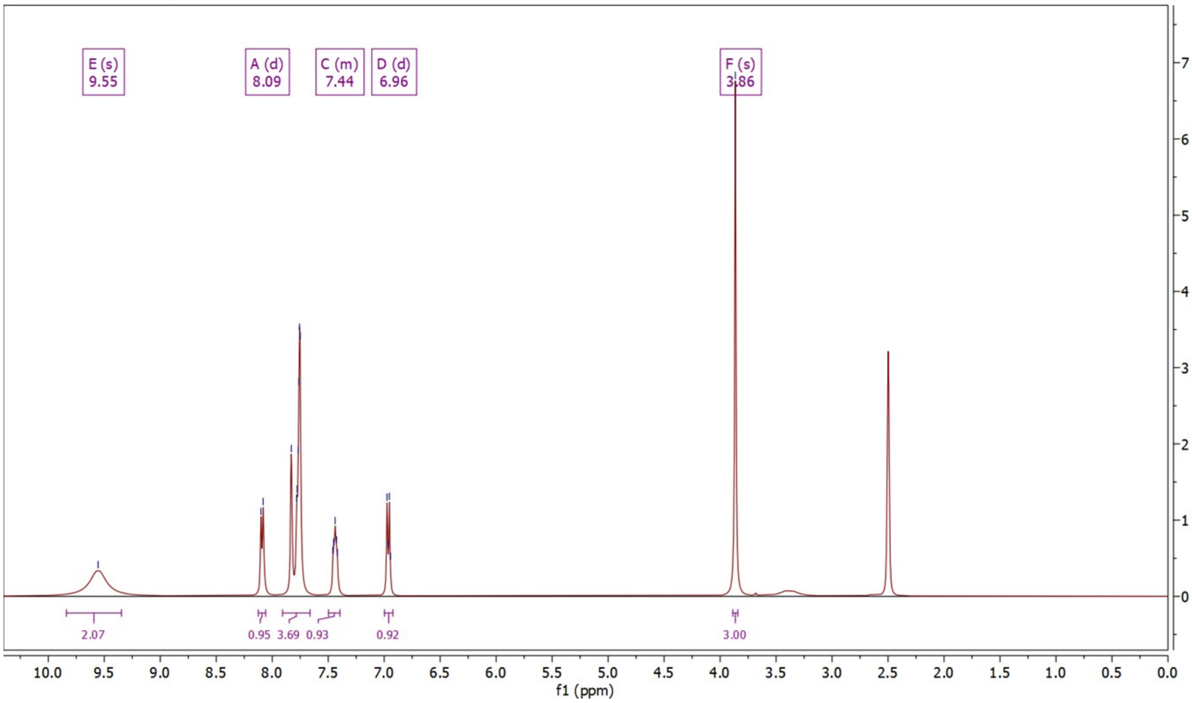

^{13}C NMR (126 MHz, DMSO-d_6)

^{13}C NMR (126 MHz, DMSO) δ 172.5, 154.3, 148.7, 147.4, 145.9, 138.0, 133.3, 124.6, 124.4, 122.2, 121.8, 121.2, 118.3, 115.5, 111.7, 55.7.

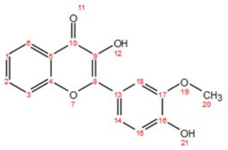

3-hydroxy-2-(4-hydroxy-3-methoxyphenyl)-4H-chromen-4-one
Chemical Formula: $C_{16}H_{12}O_5$

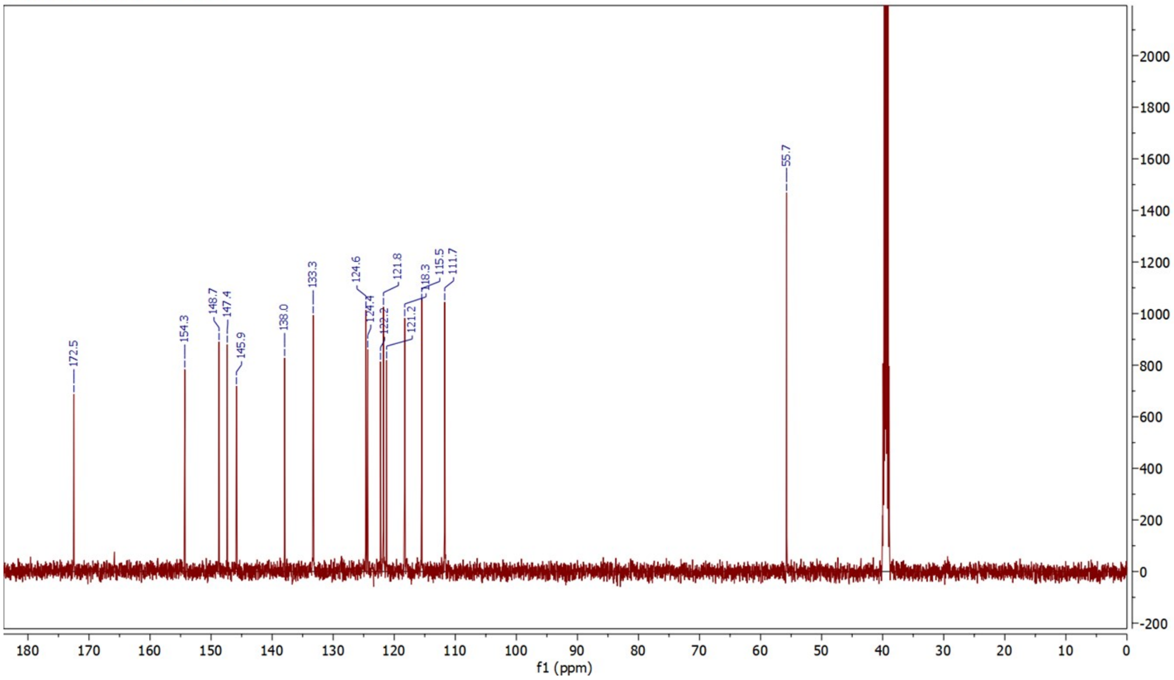

Figure S8.^{1}H NMR (*top*) and ^{13}C NMR (*bottom*) spectra for flavonol **3h**

^{1}H NMR (500 MHz, DMSO-d_6)

^{1}H NMR (500 MHz, DMSO) δ 9.47 (s, 1H), 8.09 (d, J = 7.9 Hz, 1H), 7.86 (d, J = 8.6 Hz, 1H), 7.83 – 7.74 (m, 3H), 7.45 (t, 1H), 7.14 (d, J = 8.6 Hz, 1H), 3.84 (s, 6H).

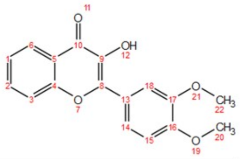

2-(3,4-dimethoxyphenyl)-3-hydroxy-4H-chromen-4-one
Chemical Formula: $C_{17}H_{14}O_5$

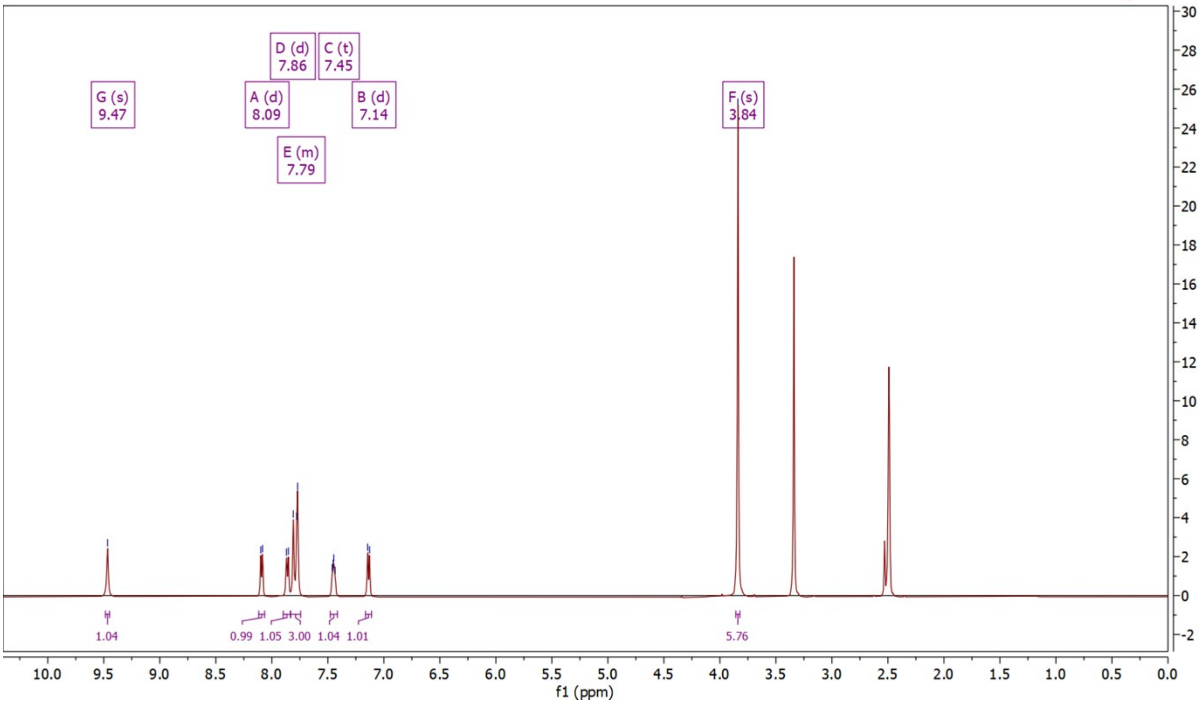

^{13}C NMR (151 MHz, DMSO-d_6)

^{13}C NMR (151 MHz, dmso) δ 173.1, 154.9, 150.8, 148.9, 145.9, 138.7, 133.9, 125.2, 124.9, 124.1, 122.0, 121.7, 118.9, 112.0, 111.5, 56.1, 56.1.

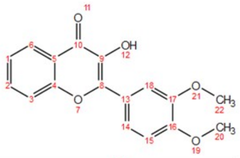

2-(3,4-dimethoxyphenyl)-3-hydroxy-4H-chromen-4-one
Chemical Formula: $C_{17}H_{14}O_5$

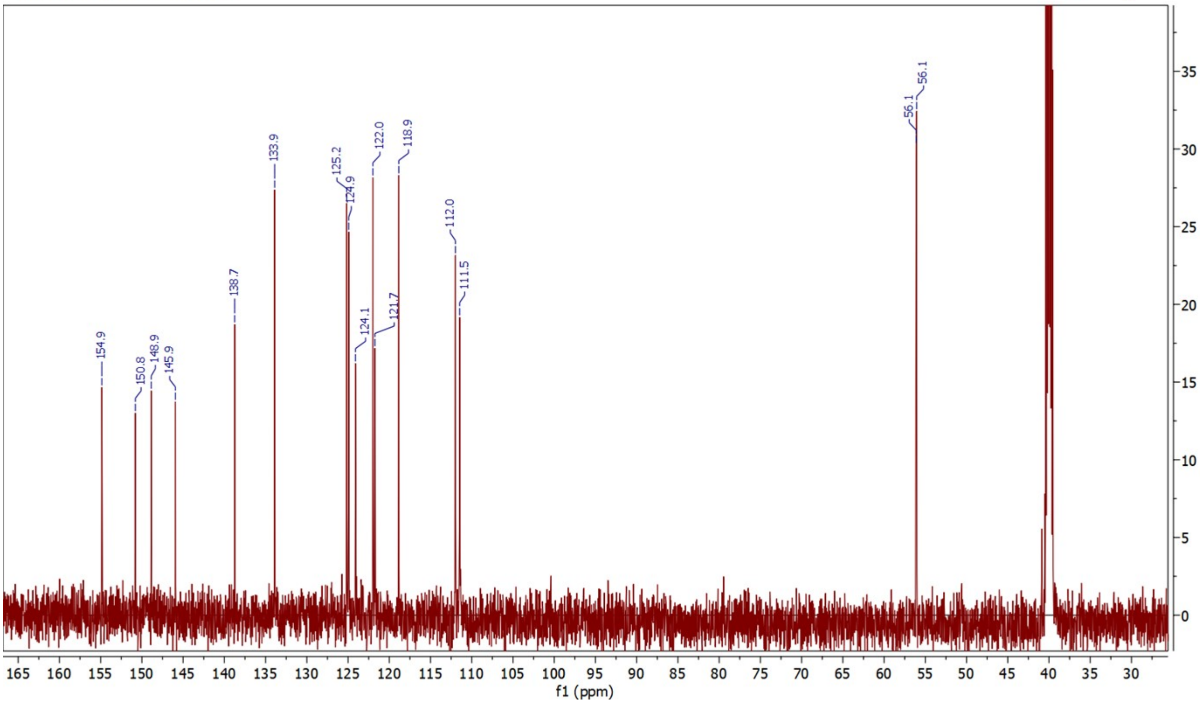

Figure S9.^{1}H NMR (*top*) and ^{13}C NMR (*bottom*) spectra for flavonol **3i**

^{1}H NMR (500 MHz, DMSO-d_6)

^{1}H NMR (500 MHz, DMSO) δ 9.48 (s, 1H), 8.12 – 8.06 (m, 1H), 7.83 (d, J = 7.1 Hz, 2H), 7.76 (d, J = 5.1 Hz, 2H), 7.50 – 7.30 (m, 5H), 7.22 (d, J = 8.8 Hz, 1H), 5.17 (s, 2H), 3.85 (s, 3H).

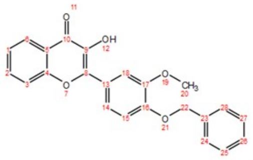

2-(4-(benzyloxy)-3-methoxyphenyl)-3-hydroxy-4H-chromen-4-one
Chemical Formula: $C_{23}H_{18}O_5$

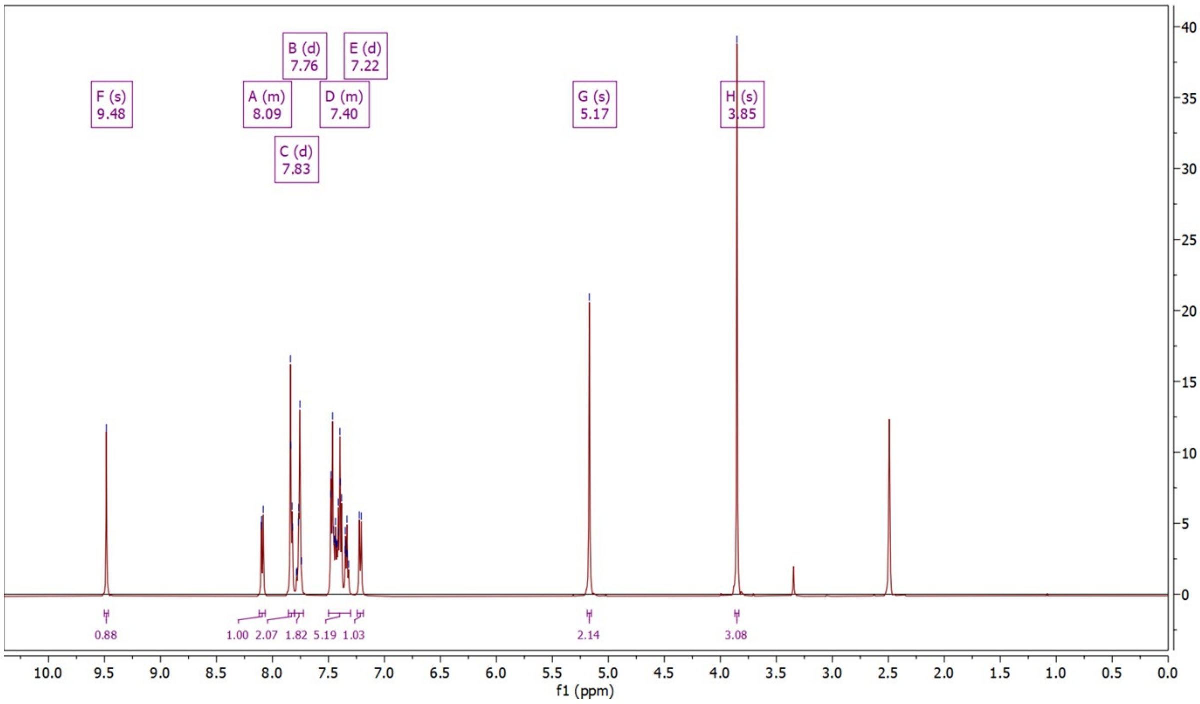

^{13}C NMR (126 MHz, DMSO-d_6)

^{13}C NMR (126 MHz, DMSO) δ 172.6, 154.4, 149.3, 148.7, 145.3, 138.3, 136.7, 133.4, 128.4, 127.9, 127.9, 124.7, 124.4, 123.9, 121.3, 121.2, 118.3, 113.0, 111.3, 69.8, 55.7.

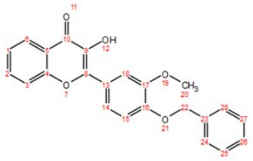

2-(4-(benzyloxy)-3-methoxyphenyl)-3-hydroxy-4H-chromen-4-one
Chemical Formula: $C_{23}H_{18}O_5$

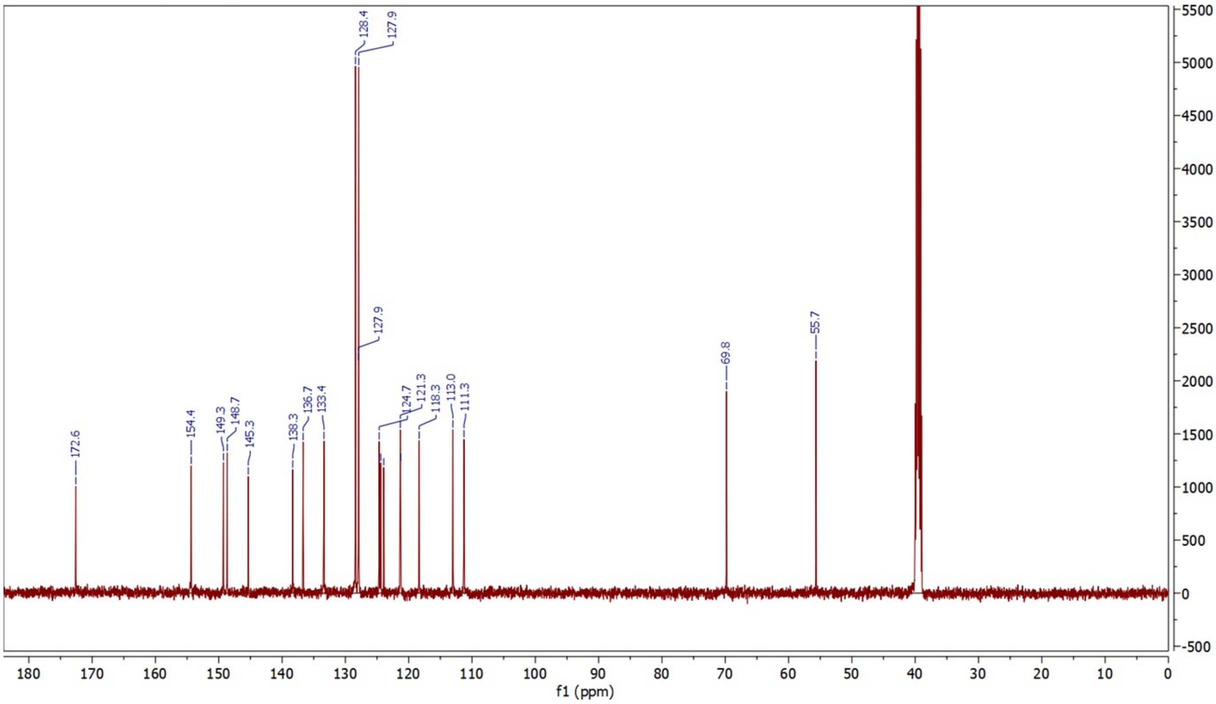

Figure S10.^{1}H NMR (*top*) and ^{13}C NMR (*bottom*) spectra for flavonol **3j**

^{1}H NMR (400 MHz, DMSO-d_6)

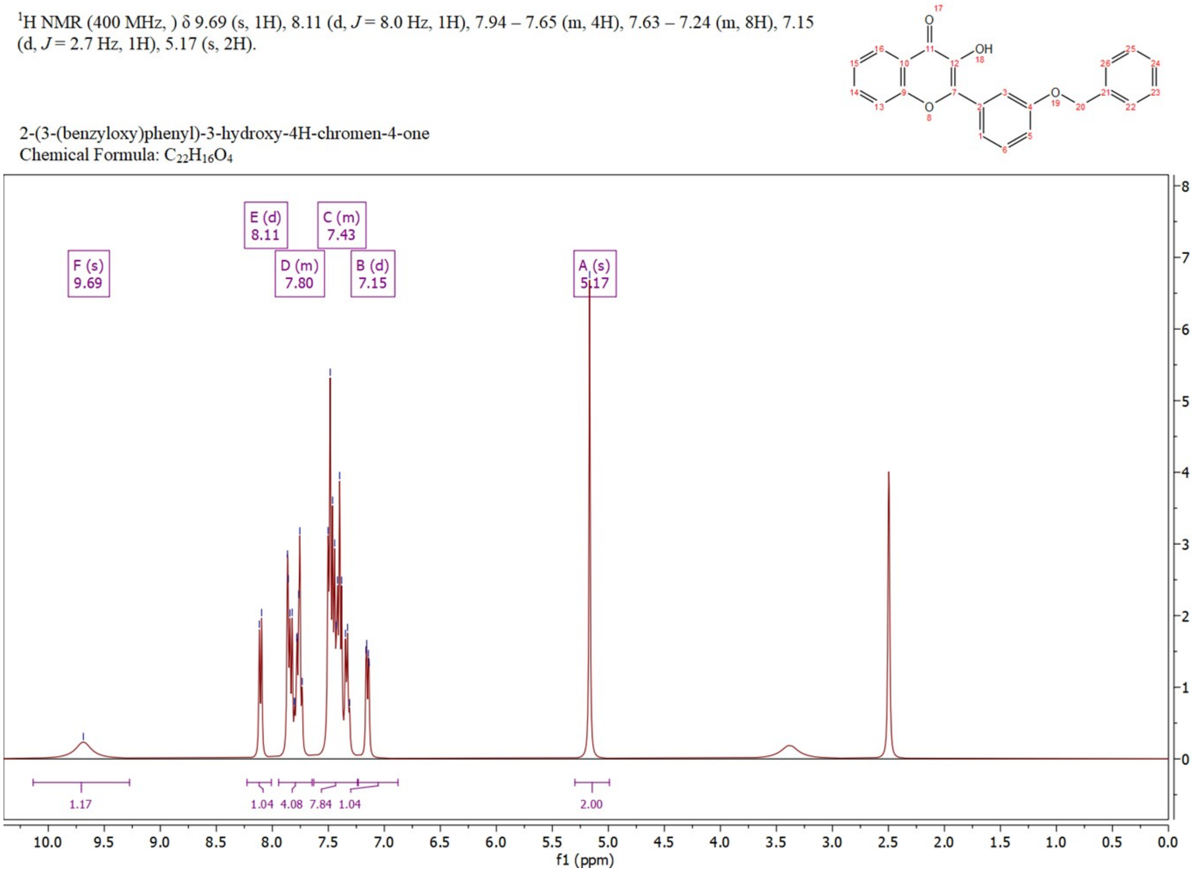

^{13}C NMR (126 MHz, DMSO-d_6)

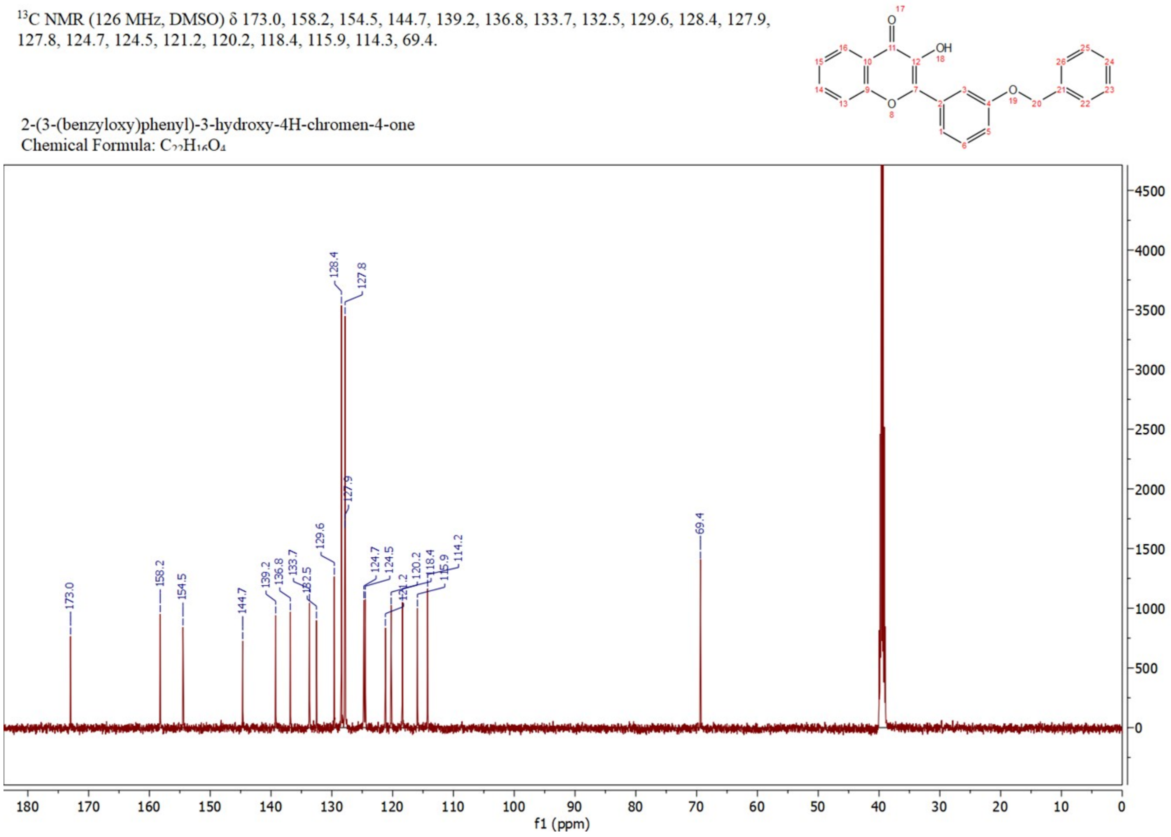

Figure S11.^{1}H NMR (*top*) and ^{13}C NMR (*bottom*) spectra for flavonol **3k**

^{1}H NMR (400 MHz, DMSO-d_6)

^{1}H NMR (400 MHz, dmso) δ 9.50 (s, 1H), 8.10 (dd, J = 8.0, 1.6 Hz, 1H), 7.94 – 7.87 (m, 2H), 7.84 – 7.72 (m, 2H), 7.60 – 7.26 (m, 6H), 7.17 (dd, 1H), 5.18 (s, 2H), 3.86 (s, 3H).

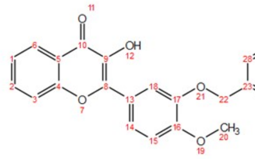

2-(3-(benzyloxy)-4-methoxyphenyl)-3-hydroxy-4H-chromen-4-one

Chemical Formula: $C_{23}H_{18}O_5$

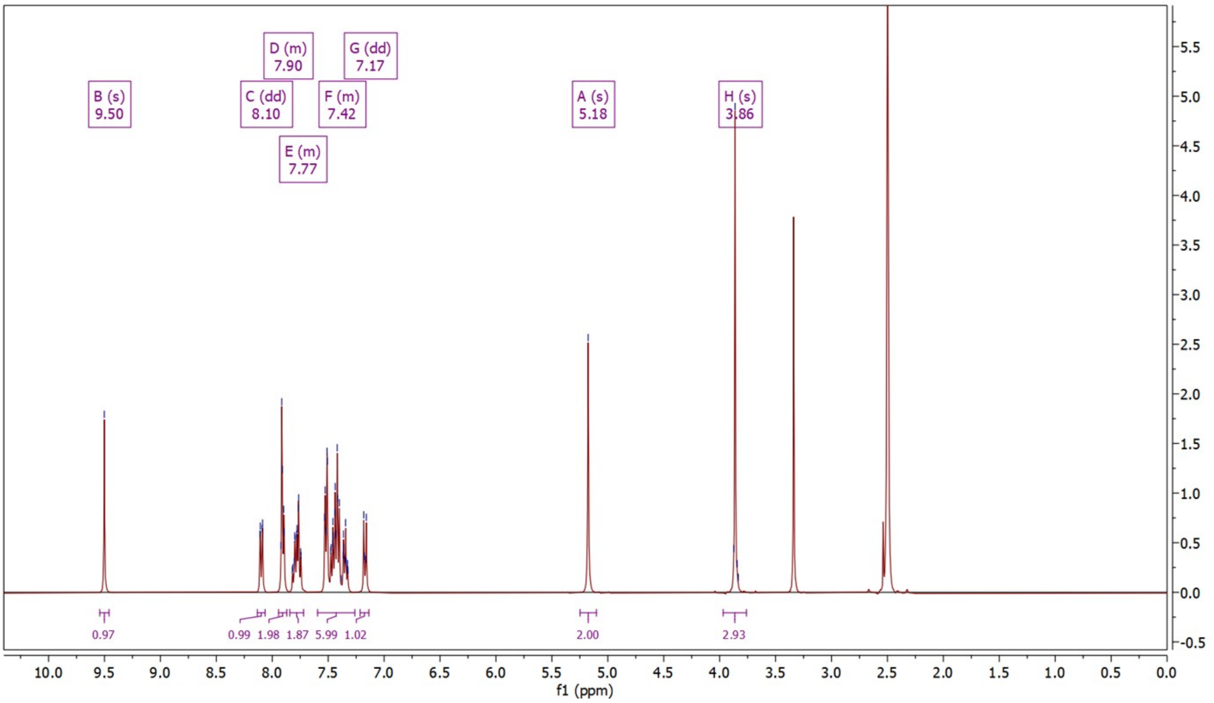

^{13}C NMR (126 MHz, DMSO-d_6)

^{13}C NMR (126 MHz, DMSO) δ 172.6, 154.3, 150.6, 147.4, 145.3, 138.3, 136.9, 133.4, 128.4, 128.1, 127.9, 124.7, 124.5, 123.5, 121.9, 121.2, 118.3, 112.7, 111.7, 70.2, 55.6.

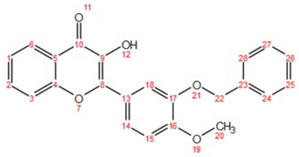

2-(3-(benzyloxy)-4-methoxyphenyl)-3-hydroxy-4H-chromen-4-one

Chemical Formula: $C_{23}H_{18}O_5$

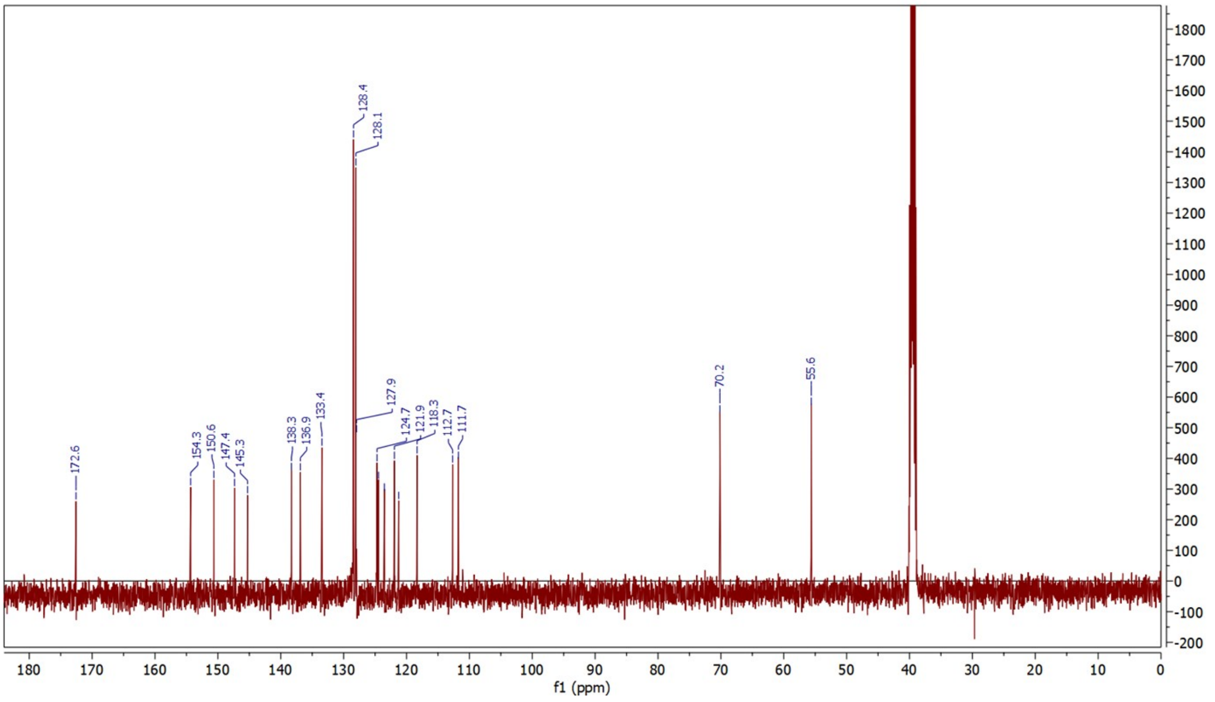

Figure S12.^{1}H NMR (*top*) and ^{13}C NMR (*bottom*) spectra for flavonol **3l**

^{1}H NMR (400 MHz, DMSO-d_6)

^{1}H NMR (400 MHz, dmso) δ 9.52 (s, 0H), 8.09 (d, J = 8.0 Hz, 0H), 7.94 (s, 0H), 7.87 (d, J = 8.6 Hz, 0H), 7.83 – 7.70 (m, 0H), 7.54 – 7.28 (m, 3H), 7.25 (d, J = 8.7 Hz, 0H), 5.22 (d, J = 7.3 Hz, 1H).

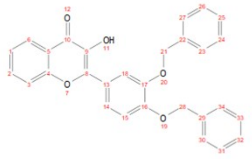

2-(3,4-bis(benzyloxy)phenyl)-3-hydroxy-4H-chromen-4-one
Chemical Formula: $C_{29}H_{22}O_5$

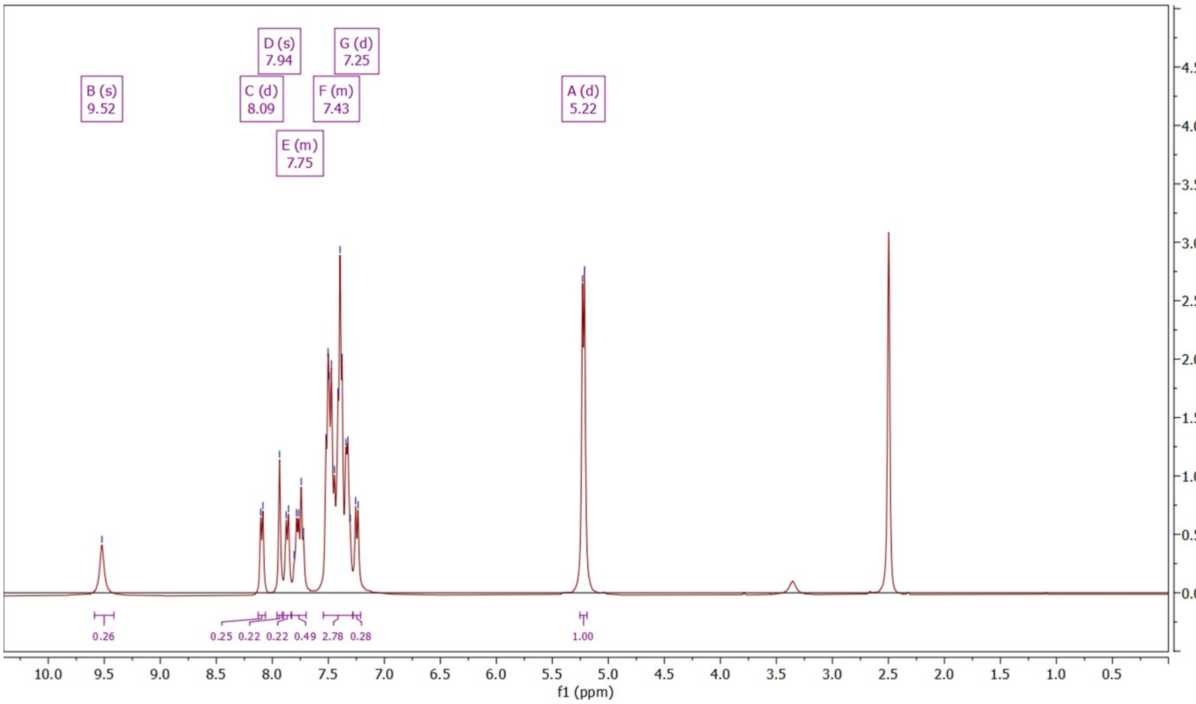

^{13}C NMR (126 MHz, DMSO-d_6)

^{13}C NMR (126 MHz, DMSO) δ 172.58, 154.33, 149.76, 147.73, 145.15, 138.36, 137.03, 136.81, 133.44, 128.40, 127.86, 127.78, 127.61, 124.68, 124.43, 123.97, 121.90, 121.24, 118.29, 113.72, 70.41, 69.87.

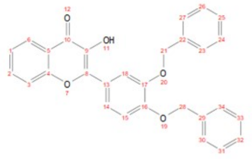

2-(3,4-bis(benzyloxy)phenyl)-3-hydroxy-4H-chromen-4-one
Chemical Formula: $C_{29}H_{22}O_5$

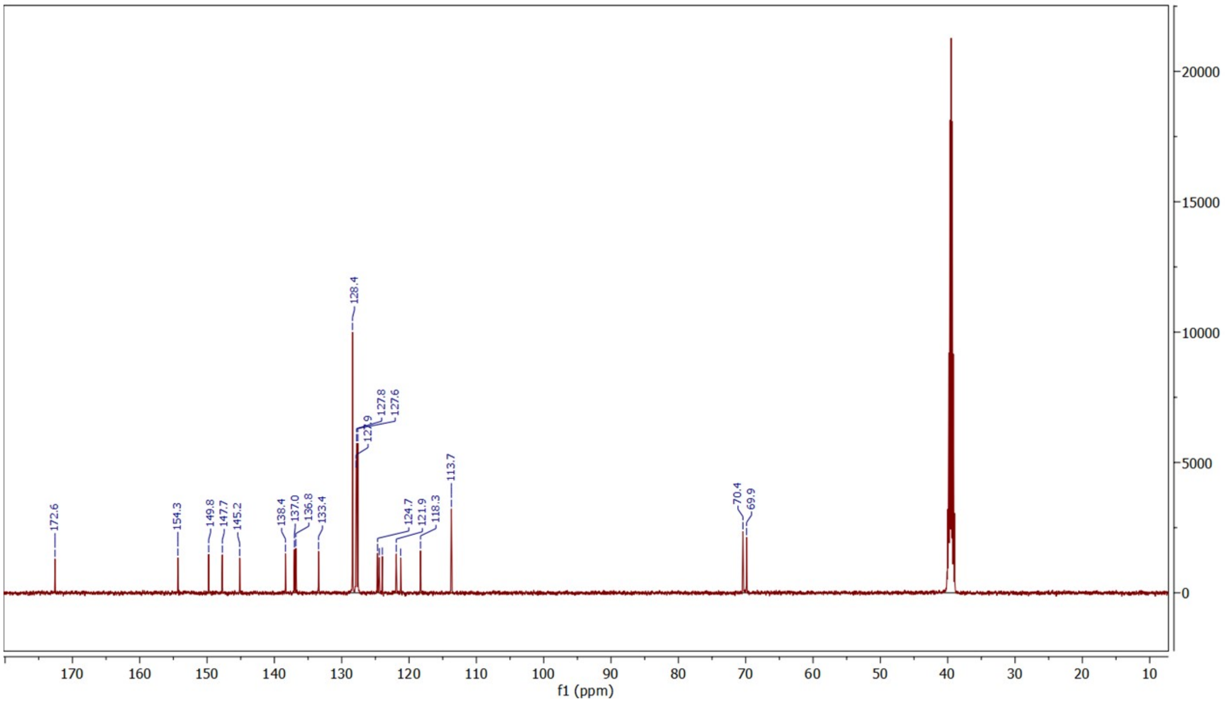

Figure S13.^{1}H NMR (*top*) and ^{13}C NMR (*bottom*) spectra for flavonol **3m**

Mass spectra of flavonol 3a-m

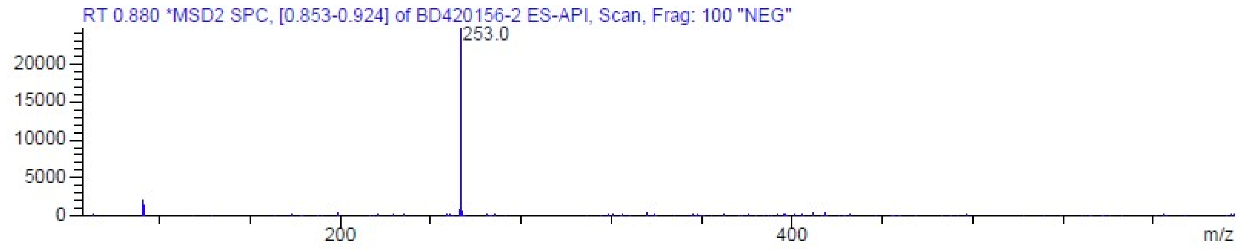

Figure S14. Mass spectrum **3a**

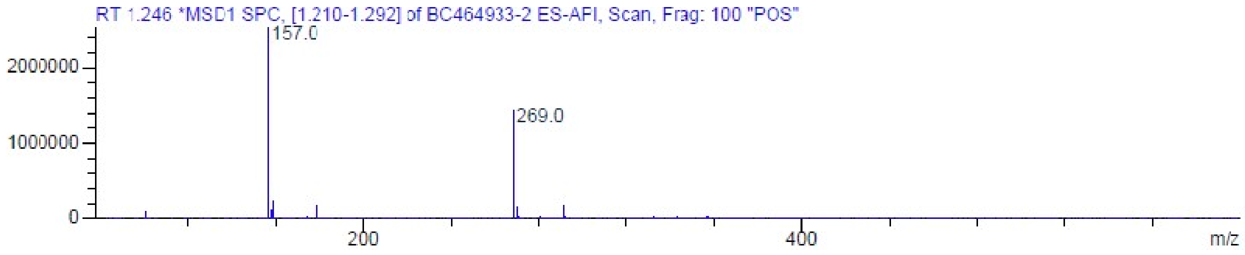

Figure S15. Mass spectrum **3b**

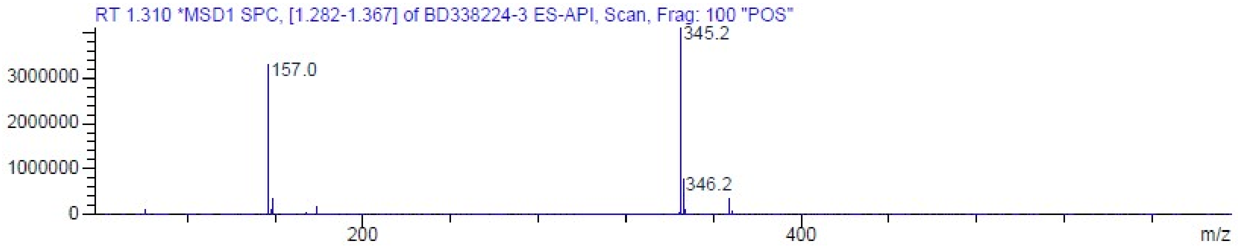

Figure S16. Mass spectrum **3c**

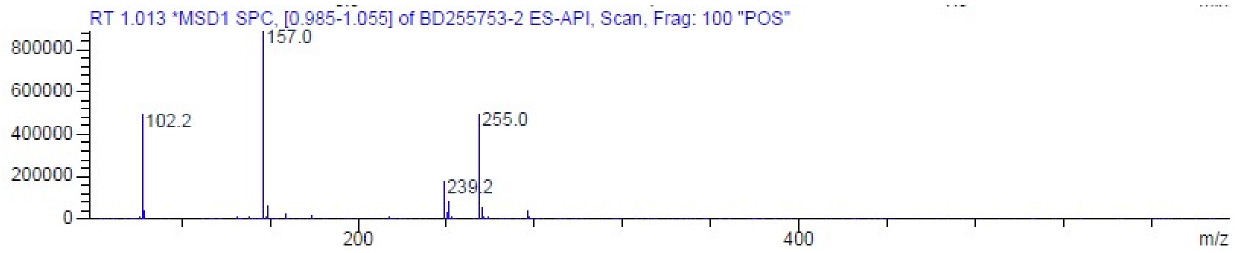

Figure S17. Mass spectrum **3d**

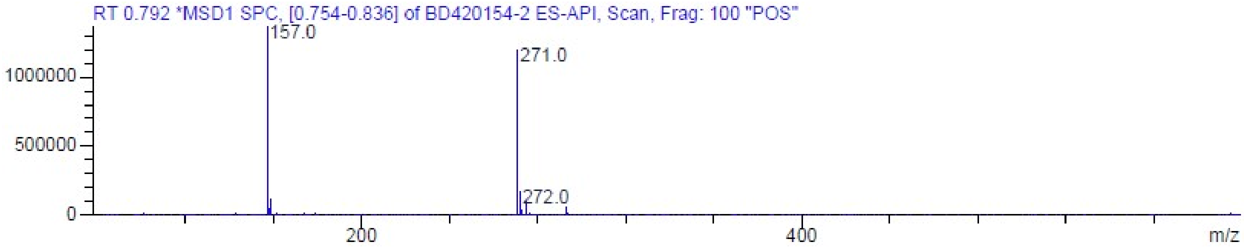

Figure S18. Mass spectrum **3e**

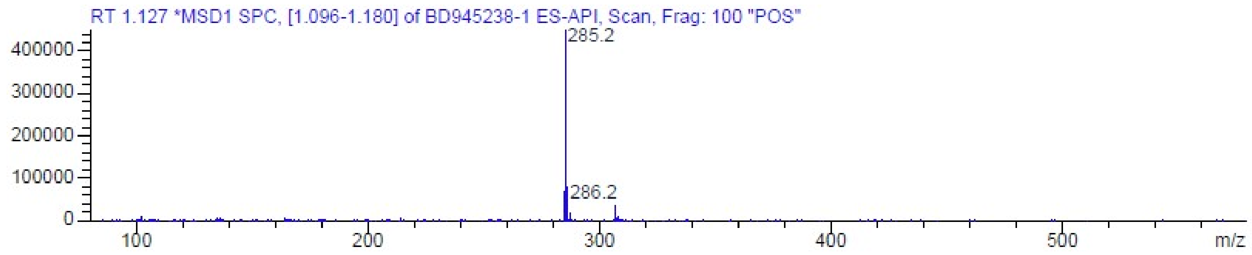

Figure S19. Mass spectrum **3f**

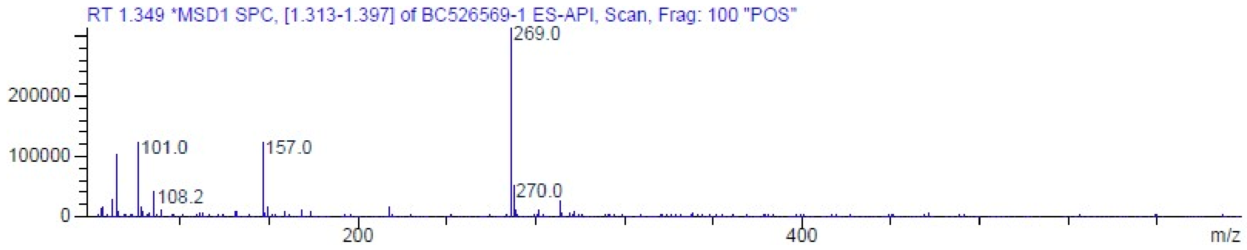

Figure S20. Mass spectrum **3g**

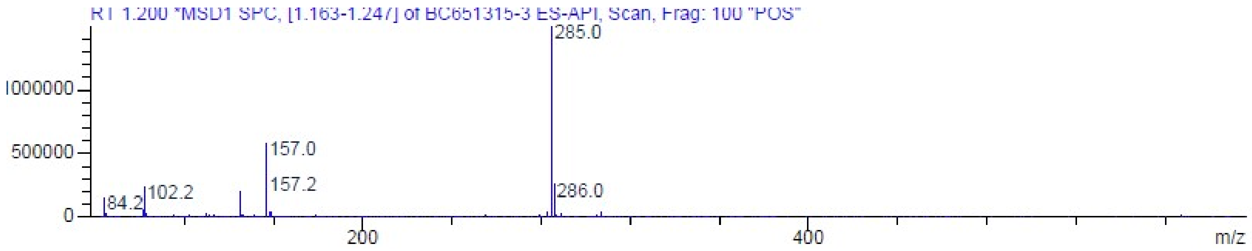

Figure S21. Mass spectrum **3h**

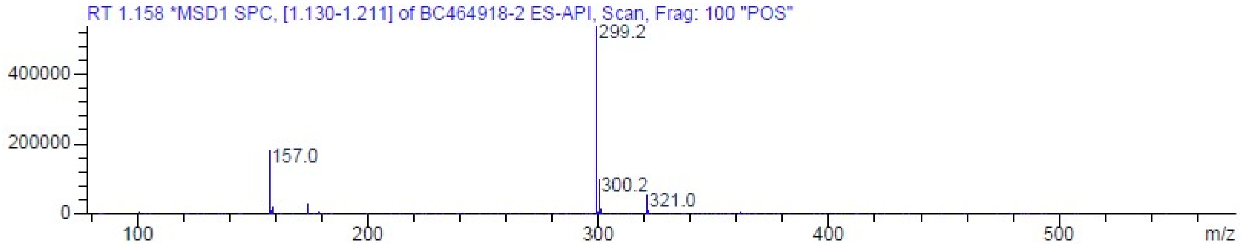

Figure S22. Mass spectrum **3i**

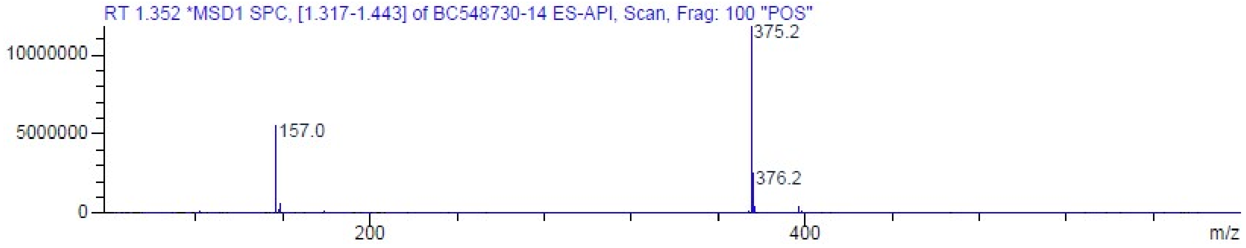

Figure S23. Mass spectrum **3j**

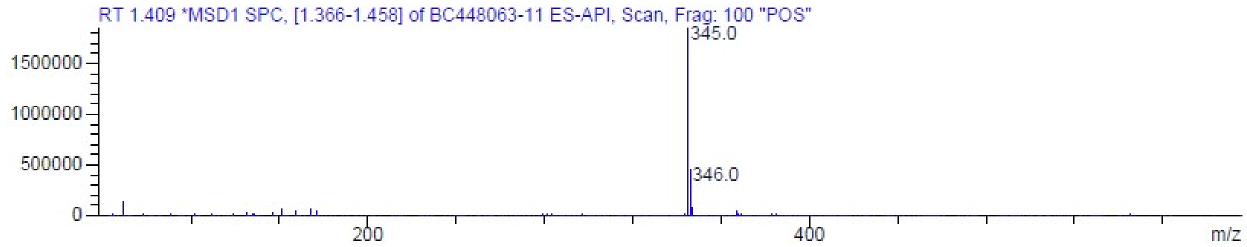

Figure S24. Mass spectrum **3k**

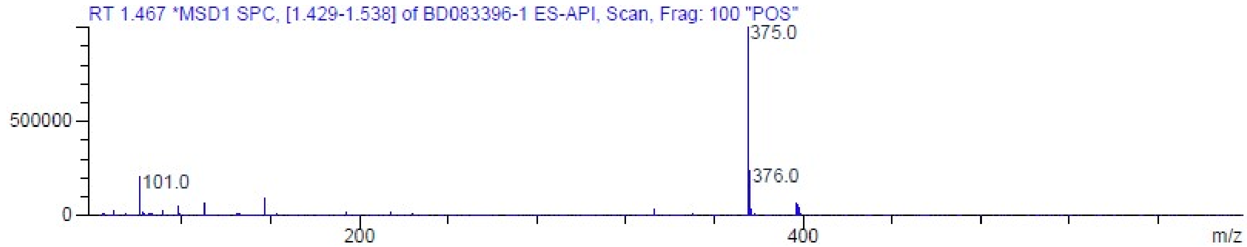

Figure S25. Mass spectrum **3l**

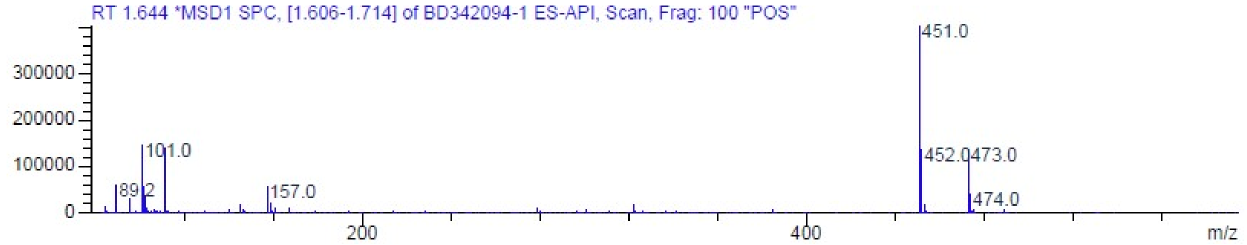

Figure S26. Mass spectrum **3m**
